# *Astragaloside IV* derivative HHQ16 ameliorates infarction-induced hypertrophy and heart failure through degradation of *lncRNA4012/9456*

**DOI:** 10.1038/s41392-023-01660-9

**Published:** 2023-10-19

**Authors:** Jingjing Wan, Zhen Zhang, Chennan Wu, Saisai Tian, Yibei Zang, Ge Jin, Qingyan Sun, Pin Wang, Xin Luan, Yili Yang, Xuelin Zhan, Lingyu Linda Ye, Dayue Darrel Duan, Xia Liu, Weidong Zhang

**Affiliations:** 1 School of Pharmacy, Second Military Medical University, Shanghai, PR China; 2 China Institute of Pharmaceutical Industry, Shanghai, PR China; 3 Key Laboratory of Medical Immunology and Institute of Immunology, Second Military Medical University, Shanghai, PR China; 4 Institute of Interdisciplinary Integrative Medicine Research, Shanghai University of Traditional Chinese Medicine, Shanghai, PR China; 5 China Regional Research Centre, International Centre of Genetic Engineering & Biotechnology, Taizhou, PR China; 6 State Key Laboratory of Medicinal Chemical Biology, College of Pharmacy, Nankai University, Tianjin, PR China; 7 Center for Phenomics of Traditional Chinese Medicine, Hospital of Traditional Chinese Medicine Affiliated to Southwest Medical University, Southwest Medical University, Luzhou, PR China; 8 Key Laboratory of Autoimmune Diseases and Precision Medicine, People’s Hospital of Ningxia Hui Autonomous Region, Yinchuan, PR China; 9 Institute of Medicinal Plant Development, Chinese Academy of Medical Sciences and Peking Union Medical College, Beijing, PR China

**Keywords:** Non-coding RNAs, Cardiology

## Abstract

Reversing ventricular remodeling represents a promising treatment for the post-myocardial infarction (MI) heart failure (HF). Here, we report a novel small molecule HHQ16, an optimized derivative of *astragaloside IV*, which effectively reversed infarction-induced myocardial remodeling and improved cardiac function by directly acting on the cardiomyocyte to reverse hypertrophy. The effect of HHQ16 was associated with a strong inhibition of a newly discovered *Egr2*-affiliated transcript *lnc9456* in the heart. While minimally expressed in normal mouse heart, *lnc9456* was dramatically upregulated in the heart subjected to left anterior descending coronary artery ligation (LADL) and in cardiomyocytes subjected to hypertrophic stimulation. The critical role of *lnc9456* in cardiomyocyte hypertrophy was confirmed by specific overexpression and knockout in vitro. A physical interaction between *lnc9456* and G3BP2 increased NF-κB nuclear translocation, triggering hypertrophy-related cascades. HHQ16 physically bound to *lnc9456* with a high-affinity and induced its degradation. Cardiomyocyte-specific *lnc9456* overexpression induced, but knockout prevented LADL-induced, cardiac hypertrophy and dysfunction. HHQ16 reversed the effect of *lnc9456* overexpression while lost its protective role when *lnc9456* was deleted, further confirming *lnc9456* as the bona fide target of HHQ16. We further identified the human ortholog of *lnc9456*, also an *Egr2*-affiliated transcript, *lnc4012*. Similarly, *lnc4012* was significantly upregulated in hypertrophied failing hearts of patients with dilated cardiomyopathy. HHQ16 also specifically bound to *lnc4012* and caused its degradation and antagonized its hypertrophic effects. Targeted degradation of pathological increased *lnc4012/lnc9456* by small molecules might serve as a novel promising strategy to regress infarction-induced cardiac hypertrophy and HF.

## Introduction

Ventricular remodeling post-myocardial infarction (MI) is the most common cause of heart failure (HF).^1^ The loss of myocardium results in an abrupt increase in the loading that initiates a neurohumoral cascade and induces a unique pattern of cardiac remodeling, characterized by the global dilatation and remote non-infarcted cardiac hypertrophy. The remote hypertrophy parallels cardiac dysfunction during post-MI remodeling, and is an independent risk factor for the development of HF.^1–5^ The restoration of myocyte size and chamber geometry to a more normal level is associated with an improvement of cardiac function, as well as many beneficial changes in molecular, metabolic, and extracellular matrix properties of the myocardium. This process is called remodeling reversibility, representing a promising treatment for the post-MI HF.^6,7^

In the past decades, numerous clinical studies have shown that the regression of reactive cardiac hypertrophy of adverse remodeling with existing pharmacological (ACE inhibitors, β-blockers, calcium-channel blockers, ANRIs, SGLT2 antagonists, etc.) or surgical therapies (aortic valve replacement, cardiac resynchronization therapy and left ventricular assist device therapy) is unequivocally linked to improved cardiac function and outcomes in both ischemic (post-MI) and non-ischemic HF populations.^8–10^ Patients without hypertrophy regression after HF therapies have a poor quality of life and increased mortality compared to those who have reverse remodeling.^11,12^ Experimental studies in infarction-induced cardiac remodeling and HF also showed consistent results,^13–15^ further supporting the benefits from hypertrophy inhibition in MI-induced HF. Therefore, targeting at hypertrophy regression may be a potential therapeutic strategy for ventricular remodeling and HF.^8–10^

The infarction-induced cardiac hypertrophy is triggered mainly by the local or systemic neuroendocrine hormones such as angiotensin II (Ang II), endothelin 1 (ET-1) or catecholamine, which activates the membrane-bound receptors and stimulates multiple downstream signaling pathways such as mitogen-activated protein kinase (MAPK), protein kinase C (PKC) and calcineurin, ultimately affects transcriptional regulatory factors for the cardiac hypertrophy genes.^1,2,16,17^ That’s why neurohormonal blockade has been an effective pharmacological therapy for the regression of cardiac hypertrophy and HF. However, clinical practice in HF patients is still far from satisfactory. At present, only less than 50% of patients underwent hypertrophy regression after HF treatments.^11,12^ During the past few decades, underlying intracellular mechanisms of infarction-induced cardiac hypertrophy have provided several potential therapeutic targets, including protein-coding and non-coding genes. Sodium/hydrogen exchange (NHE) is a common downstream mediator to Ang II and ET-1 in the cardiac cell, and the inhibition of NHE could significantly reduce cardiac hypertrophy and HF at 1-week and 12-week post-MI.^18^ The Raf/MAPK/ERK kinase (Mek)/extracellular signal-regulated kinases (Erk) pathway regulates hypertrophic responses to ET-1 in cardiomyocytes, while Hsp90 promotes the activation of Raf/Mek/Erk pathway and subsequent development of cardiac hypertrophy following MI via stabilization of c-Raf.^19^ Heat shock transcription factor 1 (HSF1) is a novel repressor of infarction-induced cardiac hypertrophy via modulating JAK2/STAT3 signaling.^20^ A20 is a tumor necrosis factor responsive gene, also an inhibitor of NF-κB signaling. Cardiac-specific overexpression of A20 could improve cardiac function and reduce compensatory cardiac hypertrophy in MI mice.^15^ The aldosterone-mineralocorticoid receptor (Aldo-MR) has been shown to drive cardiac remodeling after MI. MiR-181a is a novel regulator in the downstream networks of Aldo–MR pathway via direct targeting of Adamts1. Genetic miR-181 knockout led to deteriorated, while AAV9-mediated miR-181a overexpression improved, cardiac hypertrophy and cardiac function in murine MI model.^21^ Therefore, hypertrophic signaling molecules inside the myocytes are emerging as valid targets for the treatment of cardiac hypertrophy and HF.^22^ However, the molecular mechanisms for hypertrophy regression remain to be elucidated and therapies that can directly reverse an existing hypertrophy at the level of the cardiomyocyte are currently unavailable.

*Astragali Radix* is a commonly used traditional Chinese medicine in clinical practice to treat chronic HF.^23–25^ The main active ingredient of *Astragali Radix* has been confirmed to be *astragaloside IV*.^26,27^ Over the last decade, scientific evidence has shown that *astragaloside IV* significantly reversed the adverse remodeling in the experimental chronic HF, with the direct target and effect obscure.^26,28,29^ Therefore, *astragaloside IV* might provide valuable candidate compounds for new drug development. Here, we optimized a novel small molecule derivative of *astragaloside IV* named HHQ16, which effectively reverses post-MI remodeling and improves cardiac function via directly acting on the cardiomyocyte to produce anti-hypertrophic effect. We also discovered a new HHQ16-regulated *Egr2*-affiliated transcript *lnc9456* and its human ortholog *lnc4012* in the hypertrophied failing hearts of patients with dilated cardiomyopathy (DCM), and provided novel mechanistic insights into the roles of *lnc4012*/*lnc9456* in the development of cardiac hypertrophy and HF. HHQ16 effectively reversed infarction-induced hypertrophy and HF by targeted degrading *lnc4012/lnc9456* with high-affinity binding and antagonizing their effects on G3BP2/NF-κB signaling. Targeted degradation of pathological increased *lnc4012/lnc9456* by small molecules might serve as a novel promising strategy to regress, possibly not merely MI-induced, cardiac hypertrophy and HF.

## Results

### A new small molecule HHQ16 significantly improves cardiac function and reverses myocardial remodeling in infarction-induced HF mice

**Fig. 1:**
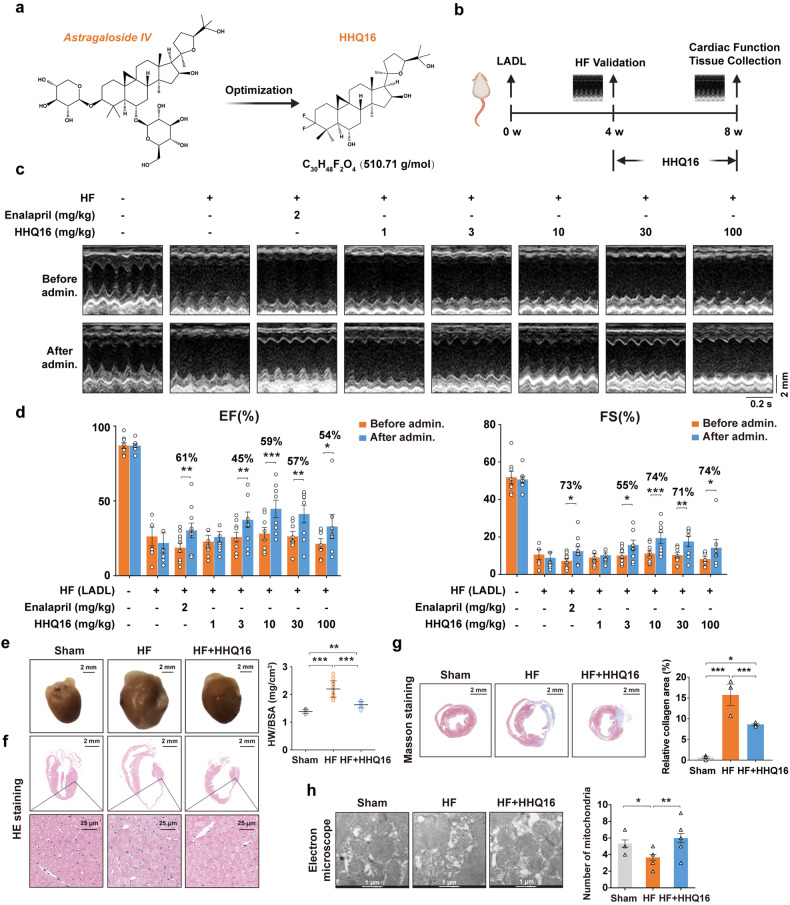
New molecule HHQ16 significantly improves cardiac function and reverses myocardial remodeling in HF mice. **a** Chemical structure of *astragaloside IV* and its derivative HHQ16. **b** Schematic overview of the experimental design in the mice model. Representative M-mode images (**c**) and statistical analysis of EF (%) and FS (%) (**d**) from mice underwent sham or LADL surgery for 4 weeks, then treated by daily intragastric administration of vehicle, enalapril, or HHQ16 at the doses indicated for 4 weeks. The cardiac function was detected by echocardiography at 4 (before admin.) and 8 (after admin.) weeks post-LADL (*n* = 7–10). **e** Representative photographs of mouse heart (left) and statistical analysis of heart weight to body surface area ratio (HW/BSA, right). Mice were treated as mentioned in **b** with the dose of HHQ16 at 10 mg/kg (*n* = 12–18). **f** Representative H&E of the myocardial tissues derived from mice treated as mentioned in **e** (*n* = 3). **g** Representative Masson’s trichrome staining (left) and quantification (right) of myocardial tissues in mice treated as mentioned in **e** (*n* = 3). **h** Representative electron microscopic images (left) and their quantification (right) of mitochondrial number in the myocardial tissues derived from mice treated as mentioned in **e** (*n* = 6). Data are presented as the means ± SEM. *P < 0.05, **P < 0.01, ***P < 0.001 by two-way ANOVA with Sidak’s multiple comparisons test (**d**) and one-way ANOVA with Tukey’s multiple comparisons test (**e**, **g**, **h**)

We used *astragaloside IV* as a lead compound and screened a new small molecule derivative HHQ16 (Fig. 1a). We then investigated the effects of HHQ16 on HF mice caused by the left anterior descending coronary artery ligation (LADL)-induced ischemia (Fig. 1b). As shown in Fig. 1c, d, the left ventricular ejection fraction (LVEF) and left ventricular fractional shortening (LVFS) of the mice were significantly reduced 4 weeks after LADL surgery when compared with that of the mice in the sham control group, suggesting that LADL had compromised the cardiac function.^30^ These mice were subsequently treated by daily gavage with control solvent or HHQ16 at different doses of 1, 3, 10, 30 and 100 mg/kg for another 4 weeks (Fig. 1b). Enalapril (ACEI class) and LCZ696 (ARNI class), two first-line medicines for the treatment of HF, were chosen as positive controls. At the end of the 8th week after LADL the LVEF and LVFS of the mice subjected to control solvent decreased further compared to that at the 4th week, indicating a progressive development of HF. In contrast, the LVEF and LVFS of the mice receiving HHQ16 significantly increased in a dose-dependent manner (Fig. 1d). HHQ16 at 10 mg/kg showed the best effect in the improvement of LVEF and LVFS, a comparable effect to that of enalapril (2 mg/kg) but better than that of LCZ696 (100 mg/kg, Supplementary Fig. 1).

Meanwhile, HHQ16 significantly reduced the enlarged heart size and the heart weight to body surface area ratio (HW/BSA) (Fig. 1e). H&E and Masson staining revealed that HHQ16 reversed significantly the LADL-induced hypertrophy by decreasing the cell volume (size) and slippage (disorderly aligned myocytes) (Fig. 1f), as well as fibrosis (Fig. 1g). The mitochondrial morphology was also recovered by HHQ16 as observed under electron microscopy (Fig. 1h). Taken together, these results provided compelling evidence that HHQ16 treatment could effectively reverse the LADL-induced deterioration of cardiac function and structural remodeling in mice.

### HHQ16 acts directly on cardiomyocytes to produce anti-hypertrophic effect

**Fig. 2:**
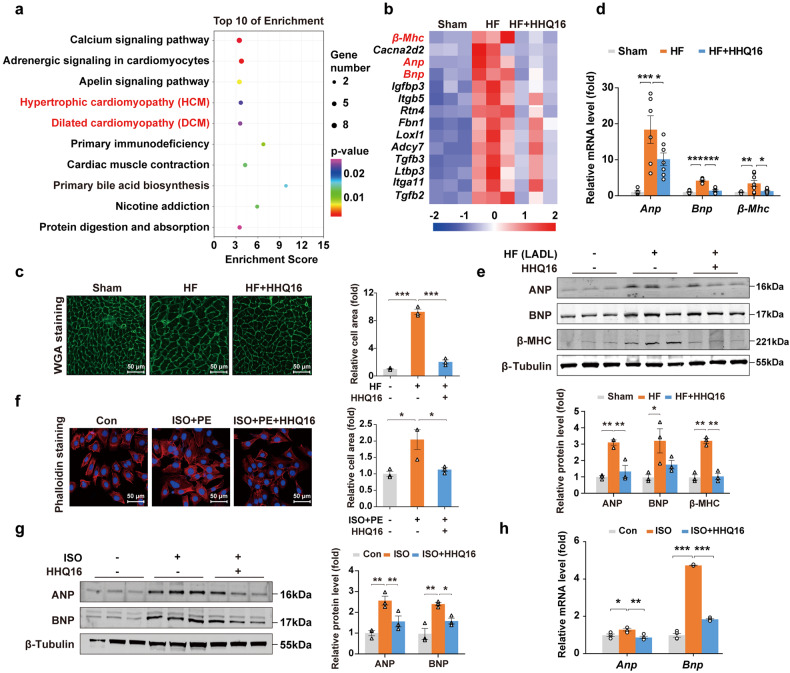
HHQ16 restrains cardiac hypertrophy by directly acts on cardiomyocytes. **a** The top 10 of KEGG analysis from the RNA-sequencing (mRNA) of myocardial tissues derived from mice treated as mentioned in Fig. 1e. **b** Red-labeled KEGG items in **a** were further enriched. Blue square: downregulated genes; Red square: upregulated genes. **c** Representative WGA staining (left) and its quantification (right) in myocardial tissue derived from mice treated as mentioned in Fig. 1e (*n* = 3). **d** qRT-PCR detection for hypertrophy biomarkers *Anp*, *Bnp*, and β-*Mhc* in myocardial tissues of mice treated as mentioned in Fig. 1e (*n* = 6–8). **e** Western blotting (upper) and its quantification (lower) for ANP, BNP, and β-MHC in myocardial tissues of mice treated as mentioned in Fig. 1e (*n* = 3). **f** Representative phalloidin staining (left) and its quantification (right) of HL-1 mouse cardiomyocytes treated with vehicle or ISO + PE (100 μM each) in the absence or presence of HHQ16 (100 nM) for 24 h (*n* = 3). **g** Western blotting (left) and its quantification (right) for ANP and BNP of neonatal mouse primary cardiomyocytes treated with vehicle or ISO (100 μM) in the absence or presence of HHQ16 (100 nM) for 24 h (*n* = 3). **h** qRT-PCR detection for *Anp* and *Bnp* in neonatal mouse primary cardiomyocytes treated as mentioned in **g** (*n* = 4). Data are presented as the means ± SEM. *P < 0.05, **P < 0.01, ***P < 0.001 by one-way ANOVA with Tukey’s multiple comparisons test (**c**–**h**)

To investigate the molecular mechanisms underlying the effects of HHQ16 on HF, we used the filtering and validation strategy of the RNA-sequencing to analyze differentially expressed transcripts including protein-coding mRNAs and long non-coding RNAs (*lncRNAs*). We found that the differential mRNAs affected by HHQ16 were mainly enriched in dilated cardiomyopathy and hypertrophic cardiomyopathy (Fig. 2a, and Supplementary Fig. 2a–d), as well as hypertrophic marker *β-Mhc*, *Anp* and *Bnp* (Fig. 2b), implicating that the small molecule HHQ16 has a prominent regulatory effect on cardiac hypertrophy. WGA staining revealed that HHQ16 reduced significantly the LADL-induced hypertrophy by decreasing the cell volume (size) (Fig. 2c). Further at the transcriptional and protein levels, the LADL-induced increase in ANP, BNP, and β-MHC were also reversed by HHQ16 (Fig. 2d, e).

MI-induced cardiomyocytes hypertrophy in the remote area is driven mainly by the neuroendocrine activation.^1,2^ Therefore, consistent with the pathophysiologic mechanism of in vivo MI-induced hypertrophy model, isoproterenol (ISO), Ang II or phenylephrine (PE) stimulated cardiomyocytes were used as in vitro hypertrophy model. Phalloidin staining assay further confirmed that HHQ16 directly acted on in vitro cardiomyocytes and limited their volume expansion induced by ISO + PE stimulation (Fig. 2f) without the involvement of other cells in the cardiac tissue. HHQ16 also reversed the increase in hypertrophic expression of ANP and BNP (Fig. 2g–h) induced by ISO stimulation of primary mice cardiomyocytes, but not affect their proliferation activity (Supplementary Fig. 2e). These data suggested that hypertrophic cardiomyocytes could recover to more normal size by HHQ16 treatment, which should be responsible for the effect of HHQ16 to reverse infarction-induced remodeling and HF.

### The effect of HHQ16 was associated with a strong inhibition of a new *Egr2*-affiliated transcript *lnc9456*

**Fig. 3:**
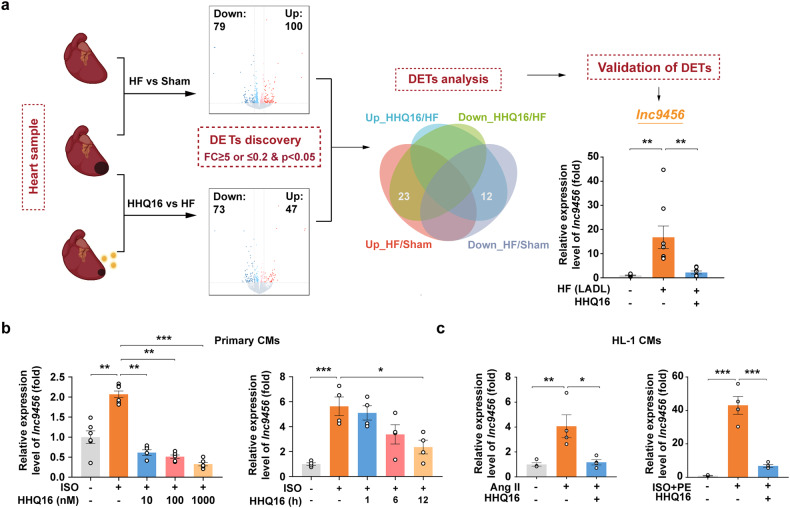
A new transcript *lnc9456* is screened out to be strongly inhibited by HHQ16. **a** The discovery procedure of *lnc9456* from the RNA-sequencing (*lncRNAs*) of myocardial tissues derived from mice treated as mentioned in Fig. 1e. Volcano plot shows the differentially expressed transcripts (DETs) discovery by using the *P* < 0.05 and foldchange (FC) ≥ 5 or ≤0.2 as the threshold of significant difference. Then, DETs analysis generates Venn diagram to identify 35 transcripts of interest. Finally, 35 transcripts were validated by qRT-PCR and *lnc9456* was screened out. **b** qRT-PCR detection for *lnc9456* in neonatal mouse primary cardiomyocytes treated with vehicle or ISO (100 μM) in the absence or presence of HHQ16 at the dose indicated for 12 h (left, *n* = 6) or at the dose of 100 nM for the time indicated (right, *n* = 4). **c** qRT-PCR detection for *lnc9456* in HL-1 mouse cardiomyocytes treated with vehicle or Ang II (50 μM, left) or ISO + PE (100 μM each, right) in the absence or presence of HHQ16 (100 nM) for 24 h. (*n* = 4). Data are presented as the means ± SEM. *P < 0.05, **P < 0.01, ***P < 0.001 by one-way ANOVA with Tukey’s multiple comparisons test (**a**–**c**)

*LncRNAs* perform crucial roles in biological processes via regulating the expression of protein-coding mRNAs.^31,32^ To further clarify the mechanism underlying the transcriptional inhibition of HHQ16 in hypertrophic marker *β-Mhc*, *Anp* and *Bnp*, *lncRNAs* sequencing data was further analyzed (Supplementary Fig. 3a, b). By using the *P* < 0.05 and foldchange (FC) ≥ 5 or ≤0.2 as the threshold of significant difference, a total of 35 *lncRNAs* in mouse hearts were identified, including 12 down-regulated *lncRNAs* in HF which were up-regulated by HHQ16, and 23 up-regulated *lncRNAs* in HF which were down-regulated by HHQ16 (Fig. 3a, middle). These results were further validated by qRT-PCR in LADL-treated mice and ISO- or Ang II- stimulated primary mouse cardiomyocytes, and *ENSMUST00000219456* (*lnc9456*) was finally screened out as the most affected *lncRNA* by HHQ16 (Supplementary Fig. 3c). According to the Ensemble database, *lnc9456* is the only transcript of the gene *GM32255* located upstream of *Egr2* gene on chromosome 10 (Supplementary Fig. 3d) with currently unknown function. The exact sequence of the transcript was determined by 5’ and 3’ rapid amplification of complementary DNA ends (RACE) as a 1365 nt *lncRNA* (Supplementary Table 1).

As shown in Fig. 3a, *lnc9456* was significantly increased by approximately 17-fold in the hypertrophied hear, which was drastically inhibited by HHQ16 (Fig. 3a, right). HHQ16 also significantly inhibited ISO, Ang II or ISO + PE induced high expression of *lnc9456* in the primary or HL-1 cardiomyocytes (Fig. 3b, c), implicating that the effects of HHQ16 on cardiac hypertrophy and HF is associated with a strong inhibition of a new *Egr2*-affiliated transcript *lnc9456*.

### *Lnc9456* is critical for cardiomyocyte hypertrophy

**Fig. 4:**
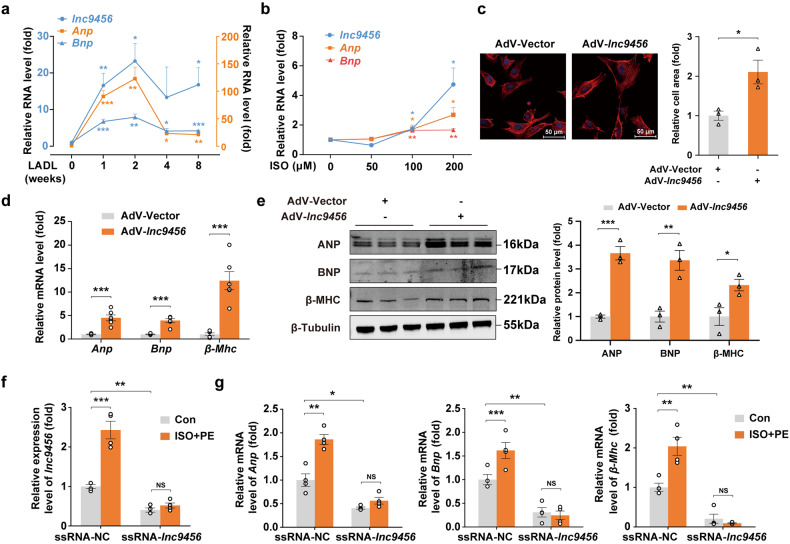
*Lnc9456* is critical for cardiomyocyte hypertrophy. **a** Synchronous expression of *lnc9456* and hypertrophic biomarkers *Anp* or *Bnp* of myocardial tissues from mice after LADL surgery were detected by qRT-PCR at the indicated time points. (*n* = 6–10). **b** qRT-PCR detection for *lnc9456*, *Anp* and *Bnp* in neonatal mouse primary cardiomyocytes treated by vehicle or ISO (50, 100, 200 μM) for 12 h (*n* = 6). **c** Representative phalloidin staining (left) and its quantification (right) of HL-1 mouse cardiomyocytes transfected with control adenovirus (AdV-Vector) or *lnc9456* overexpression adenovirus (AdV-*lnc9456*) for 48 h (*n* = 3). **d** qRT-PCR detection for hypertrophic biomarkers *Anp*, *Bnp* and *β-Mhc* of HL-1 mouse cardiomyocytes treated as mentioned in **c** (*n* = 6). **e** Western blotting (left) and its quantification (right) for ANP, BNP and β-MHC of HL-1 mouse cardiomyocytes treated as mentioned in **c** (*n* = 3). qRT-PCR detection for *lnc9456* (**f**) and hypertrophic biomarkers *Anp*, *Bnp*, and *β-Mhc* (**g**) in HL-1 mouse cardiomyocytes. The cells were transfected with smart silencer RNA of *lnc9456* (ssRNA-*lnc9456*) or its negative control (ssRNA-NC) for 48 h and then treated with vehicle or ISO + PE (100 μM each) for another 24 h (*n* = 4). Data are presented as the means ± SEM. *P < 0.05, **P < 0.01, ***P < 0.001 by one-way ANOVA with Dunnett’s multiple comparisons test (**a**, **b**), Student’s *t* test (**c**–**e**) and two-way ANOVA with Sidak’s multiple comparisons test (**f**, **g**)

*Lnc9456* is uniformly distributed in the cytoplasm and nucleus of cardiomyocytes (Supplementary Fig. 4a). Interestingly, it is barely detectable in normal heart, but highly expressed in hypertrophied and failing mouse hearts subjected to the LADL (Fig. 4a, and Supplementary Fig. 4b, c) in vivo, and hypertrophic cardiomyocytes treated with ISO in vitro (Fig. 4b). Under these pathological stresses, *lnc9456* was consistently upregulated and accompanied with a parallel increase in the expression of hypertrophy biomarkers such as ANP and BNP (Fig. 4a, b), suggesting that *lnc9456* might be a pathogenic factor that drives cardiomyocytes hypertrophy.

To determine functional roles of *lnc9456* in cardiomyocyte hypertrophy, we first employed recombinant adenovirus (AdV) to overexpress *lnc9456* in HL-1 mouse cardiomyocytes in vitro (Supplementary Fig. 4d). Compared to the negative control (AdV-Vector), the cardiomyocytes infected with AdV6-*lnc9456* had significantly larger cell size (Fig. 4c) and higher expression of hypertrophy biomarkers ANP, BNP and β-MHC both at transcriptional and protein level (Fig. 4d, e), indicating that the increased expression of *lnc9456* alone is sufficient to induce cardiomyocyte hypertrophy. Interestingly, when *lnc9456* knockdown with smart silencer RNA (ssRNA), the effect of ISO + PE-induced cardiomyocytes hypertrophy was abolished (Fig. 4f, g), indicating a critical role of *lnc9456* in the neurohormonal activation-driven cardiomyocytes hypertrophy.

### *Lnc9456* interacts with G3BP2 and promotes NF-κB signaling pathway

**Fig. 5:**
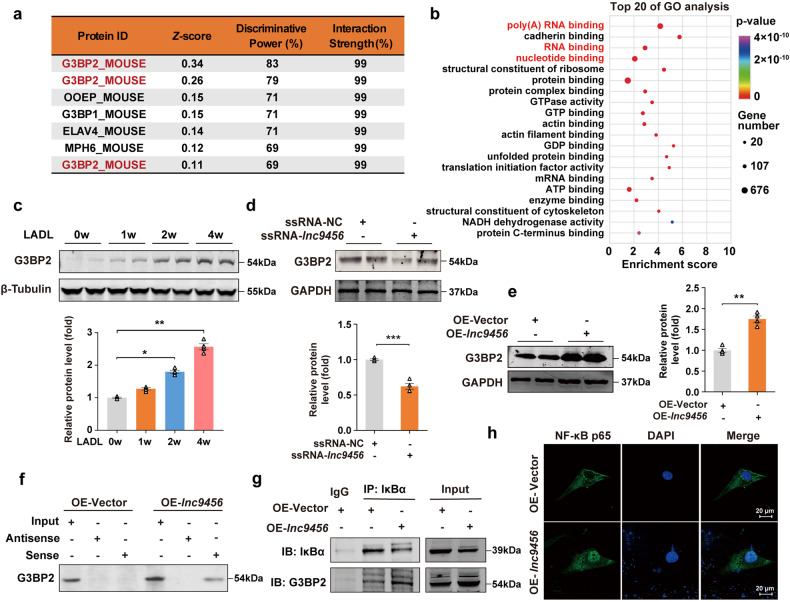
*Lnc9456* interacts with G3BP2 and promotes the nuclear translocation of p65 subunit of NF-κB. **a** The top 7 proteins that may bind to *lnc9456* were predicted by catRAPID omics module. **b** The top 20 of Gene Ontology (GO) analysis of interacting proteins pulled down by biotinylated *lnc9456* probe and identified by mass spectrometry. **c** Representative western blotting (upper) and its quantification (lower) of G3BP2 in myocardial tissues derived from mice at the indicated time post-LADL (*n* = 4). **d** Representative western blotting (upper) and its quantification (lower) of G3BP2 in HL-1 mouse cardiomyocytes transfected with smart silencer RNA of *lnc9456* (ssRNA-*lnc9456*) or its negative control (ssRNA-NC) for 72 h (*n* = 4). **e** Representative western blotting (left) and its quantification (right) of G3BP2 in HL-1 mouse cardiomyocytes transfected with control plasmid (OE-NC) or *lnc9456* overexpression plasmid (OE-*lnc9456*) for 48 h (*n* = 4). **f** RNA pull-down and western blotting detection for the binding of G3BP2 to *lnc9456* in HL-1 mouse cardiomyocytes treated as mentioned in **e**. **g** Co-IP detection for the binding of G3BP2 to IκBα in HL-1 mouse cardiomyocytes treated as mentioned in **e**. **h** Immunofluorescence staining for cellular localization of NF-κB p65 (green) in HL-1 mouse cardiomyocytes treated as mentioned in **e** (*n* = 3). Data are presented as the means ± SEM. *P < 0.05, **P < 0.01, ***P < 0.001 by one-way ANOVA with Tukey’s multiple comparisons test (**c**) and Student’s *t* test (**d**, **e**)

To explore the underlining downstream mechanisms for the effects of *lnc9456* on cardiomyocyte hypertrophy, catRAPID (http://s.tartaglialab.com/page/catrapid_group) was used to screen potential proteins that may interact with *lnc9456*. Ras-GTPase-activating protein (SH3 domain)-binding protein 2 (G3BP2) was identified with the highest probability to directly interact with *lnc9456* (Fig. 5a, and Supplementary Fig. 5a). RNA pull-down assays were performed in vitro with biotinylated *lnc9456* probe (Sense-*lnc9456*) and antisense transcript (Antisense-*lnc9456*). A total of 2113 proteins that bind to *lnc9456* were identified, and these proteins were subjected to functional GO and KEGG analyses. Poly(A) RNA binding, RNA binding and nucleotide binding were found in the top 4 of GO analysis, indicating that the main function of the protein bound to *lnc9456* was RNA binding (Fig. 5b). Further tracking down analysis showed that G3BP2 was included among these items (Fig. 5b), and cardiac muscle contraction was also enriched in KEGG analysis (Supplementary Fig. 5b).

To further verify this observation, we first examined whether LADL had any effects on the expression of G3BP2. As shown in Fig. 5c, western analysis revealed that LADL in the mice caused a time-dependent increase in G3BP2 expression, which is in parallel with the time-dependent compromised cardiac function and increase in *lnc9456* as well as hypertrophic marker ANP, BNP (Fig. 4a, and Supplementary Fig. 4c). Specifically, when the expression of *lnc9456* was interfered, the protein level of G3BP2 decreased (Fig. 5d); while in cells with high *lnc9456* expression, the protein level of G3BP2 correspondingly increased (Fig. 5e), indicating an internal relationship of *lnc9456* and G3BP2. Furthermore, our western blotting analysis revealed that the binding of the sense strand to G3BP2 was in the lysates from only the cells with *lnc9456* overexpression, but neither the sense strand nor the antisense strand binds to G3BP2 under control conditions (Fig. 5f), implicating that high level of *lnc9456* is required for the binding of G3BP2 to *lnc9456*.

Previous studies have reported that G3BP2 might be able to bind to IκBα, which in turn promotes nuclear aggregation of the NF-κB subunit p65 to initiate the hypertrophic gene transcription and induces myocardial hypertrophy.^33,34^ But it is not known why and how the binding of G3BP2 to IκBα is increased during the development of myocardial hypertrophy. Here, we confirmed that overexpression of *lnc9456* was able to potentiate the binding of G3BP2 to IκBα (Fig. 5g) by co-immunoprecipitation (Co-IP) assays. Immunofluorescence showed that *lnc9456* overexpression significantly promotes nuclear aggregation of NF-κB subunit p65 (Fig. 5h). These results strongly corroborate the notion that high level of *lnc9456* under pathological stresses increased the *lnc9456*-G3BP2 interaction and facilitated the translocation of NF-κB subunit P65 from cytoplasm to the nuclei, which activates a cascade of hypertrophy genes and exacerbates cardiac function.

### HHQ16 directly binds to *lnc9456* with high-affinity and induces its degradation

**Fig. 6:**
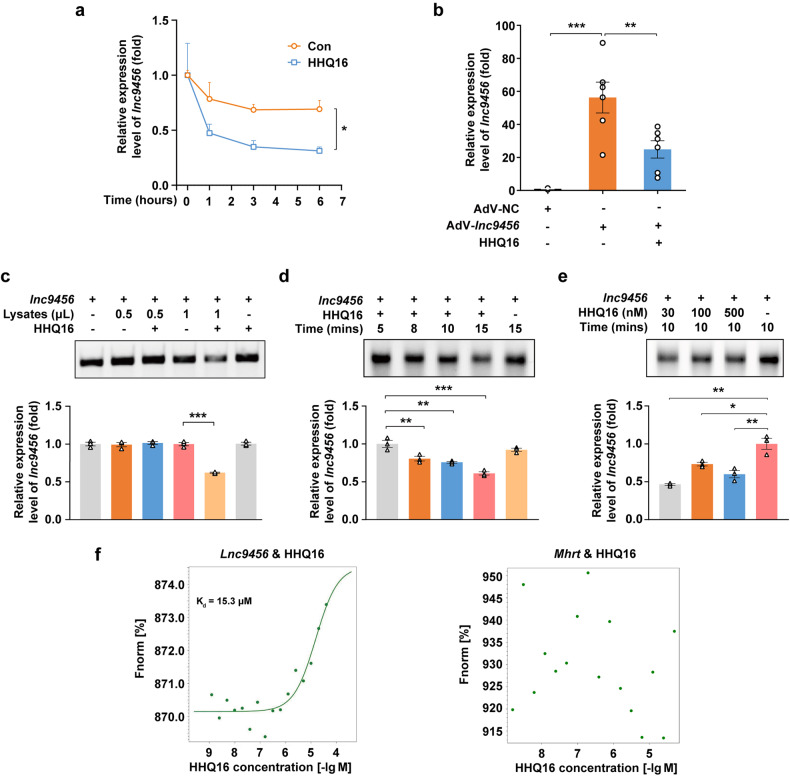
HHQ16 directly binds to *lnc9456* with high-affinity and induces its degradation. **a** qRT-PCR detection for *lnc9456* in HL-1 mouse cardiomyocytes. The cells were treated with vehicle or 100 nM of HHQ16 for 0, 1, 3 and 6 h in the presence of actinomycin D (0.5 μg/mL) (*n* = 4). **b** qRT-PCR detection for *lnc9456* in HL-1 cardiomyocytes. The cells were transfected with AdV-Vector or AdV- *lnc9456* for 24 h, then treated with vehicle or 100 nM of HHQ16 for another 24 h (*n* = 5–6). **c** Representative agarose gel electrophoresis image (upper) and its quantification (lower) of in vitro*-*transcribed *lnc9456* incubated with vehicle or HHQ16 (100 nM) in the absence or presence of indicated volume of HL-1 mouse cardiomyocytes lysates (*n* = 3). **d** Representative agarose gel electrophoresis image (upper) and its quantification (lower) of in vitro*-*transcribed *lnc9456* incubated with vehicle or HHQ16 (100 nM) for the time indicated in the presence of 1 μL of HL-1 mouse cardiomyocyte lysates (*n* = 3). **e** Representative agarose gel electrophoresis image (upper) and its quantification (lower) of in vitro*-*transcribed *lnc9456* incubated with vehicle or HHQ16 (100 nM) at the indicated doses for 10 min in the presence of 1 μL of HL-1 mouse cardiomyocyte lysates (*n* = 3). **f** MST detection for the binding affinity of HHQ16 to *lnc9456* (left) and negative control (*lncRNA* Mhrt, right). *K*_d_ value was automatically by the curve fitting. Data are presented as the means ± SEM. *P < 0.05, **P < 0.01, ***P < 0.001 by two-way ANOVA followed by Bonferroni’s post-hoc *t*-test (**a**) and one-way ANOVA with Tukey’s multiple comparisons test (**b**–**e**)

*Lnc9456* is a pro-hypertrophic factor, while HHQ16 could significantly decrease the level of *lnc9456* in hypertrophic heart and cardiomyocytes, implicating that the effect of HHQ16 on myocardial hypertrophy and HF is achieved possibly by targeting at *lnc9456*. We further explore how HHQ16 regulates the expression of *lnc9456*. Results showed that HHQ16 significantly decreased the half-time of *lnc9456* (Fig. 6a) and still had the ability to decrease AdV-*lnc9456*-induced high expression of *lnc9456* in cardiomyocytes (Fig. 6b), strongly implicating that this regulation might happen at the post-transcriptional level.

To confirm this notion, the effect of HHQ16 on the stability of *lnc9456* was further examined in vitro transcriptional system. As shown in Fig. 6c–e, HHQ16 lowered the level of in vitro transcribed *lnc9456* in a time- and dose-dependent manner (Fig. 6d, e). Importantly, the degradation of *lnc9456* by HHQ16 happened only in the presence of cardiomyocytes lysates (Fig. 6c). These prompt us to explore whether there exists an interaction between them. As expected, data from microscale thermophoresis (MST) revealed that HHQ16 had a high affinity to bind to *lnc9456* with a *K*_d_ of 15.3 μM (Fig. 6f, left), but did not show any affinity to an anti-hypertrophic *lncRNA Mhrt* (Fig. 6f, right).^35^ Thus, HHQ16 could specifically target binding to *lnc9456* and promote its degradation in cardiomyocytes.

### *Lnc9456* is essential for HHQ16 to reverse cardiac hypertrophy and dysfunction in mice

**Fig. 7:**
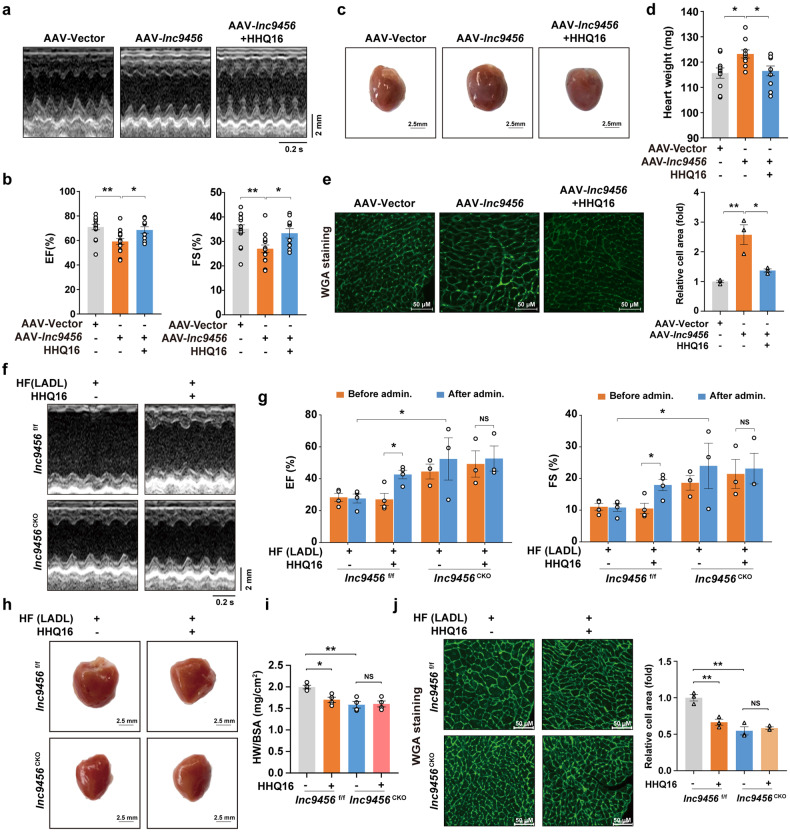
*Lnc9456* is essential for HHQ16 to reverse cardiac hypertrophy and dysfunction in mice. Representative echocardiography images (**a**) and statistical analysis of EF (%) and FS (%) (**b**) in mice. These mice were heart orthotopically injected with cardiomyocyte-specific *lnc94546* overexpression adeno-associated virus (AAV-*lnc9456*) driven by the *cTnT* promoter or its negative control (AAV-Vector). At 4th week post injection, AAV-*lnc9456* mice were treated with 10 mg/kg of HHQ16 for 2 weeks, and the cardiac function was detected by echocardiography (*n* = 10–15). Representative photographs of mouse heart (**c**) and statistical analysis of heart weight (**d**). Mice were treated as mentioned in **a** (*n* = 10). **e** Representative WGA staining (left) and its quantification (right) of mouse myocardial tissue derived from mice treated as mentioned in **a** (*n* = 3). Representative echocardiography images (**f**) and statistical analysis of EF (%) and FS (%) (**g**) from male *lnc9456*^f/f^ or *lnc9456*^CKO^ mice underwent LADL surgery for 4 weeks, then were treated with vehicle or 10 mg/kg of HHQ16 for another 4 weeks. The cardiac function was detected by echocardiography at 4 (before admin.) and 8 (after admin.) weeks post-LADL (*n* = 3–4). Representative photographs of mouse hearts (**h**) and statistical analysis of heart weight to body surface area ratio (HW/BSA, **i**) in *lnc9456*^f/f^ and *lnc9456*^CKO^ mice treated as mentioned in **f** (*n* = 3–4). **j** Representative WGA staining (left) and its quantification (right) of myocardial tissue in *lnc9456*^f/f^ and *lnc9456*^CKO^ mice treated as mentioned in **f** (*n* = 3). Data are presented as the means ± SEM. *P < 0.05, **P < 0.01, ***P < 0.001 by one-way ANOVA with Tukey’s multiple comparisons test (**b**, **d**, **e**, **i** and **j**) and two-way ANOVA with Sidak’s multiple comparisons test (**g**)

Considering that HHQ16 could degrade in vitro-transcribed *lnc9456*, we further evaluated the impact of increased *lnc9456* on the heart and the action of HHQ16 in vivo. Adeno-associated virus, AAV-*lnc9456*, driven by the specific cTNT promoter, was constructed and orthotopically injected into mouse heart to induce cardiomyocyte-specific overexpression of *lnc9456* (Supplementary Fig. 6a, left). Beginning at 4th week after the injection, HHQ16 was continuously administrated for 2 weeks. Compared to the control mice (AAV-Vector), the AAV-*lnc9456* mice had significantly increased the expression of *lnc9456* in the heart (Supplementary Fig. 6a, right), decreased LVEF and LVFS (Fig. 7a, b), larger overall heart size (Fig. 7c), higher heart weight (Fig. 7d), larger single myocyte size (Fig. 7e), and higher expression of hypertrophy biomarkers ANP, BNP and β-MHC at both transcriptional and protein levels (Supplementary Fig. 6b, c). Importantly, the hypertrophic heart was not accompanied with collagen deposition (Supplementary Fig. 6d). These data suggested that cardiomyocyte-specific overexpression of *lnc9456* alone, in the absence of MI stress, is sufficient to induce cardiac hypertrophy and dysfunction. As expected, HHQ16 treatment could decrease the high level of *lnc9456* in the heart and regress the remodeling-associated changes in these mice with cardiomyocyte-specific overexpression of *lnc9456* (Fig. 7a–e, and Supplementary Fig. 6a–d), further supporting that HHQ16 plays its role through post-transcriptional inhibition of *lnc9456*.

Meanwhile, to assess the effects of loss-of-function of *lnc9456* on the heart and HHQ16 activity in vivo, we induced the Myh6-Cre driven myocyte-specific deletion of *lnc9456* by crossing *lnc9456*^flox/flox^ mice with Myh6-Cre transgenic mice, yielding *lnc9456*^CKO^ (knockout allele) and *lnc9456*^f/f^ (control) offsprings. The *lnc9456*^f/f^ and *lnc9456*^CKO^ mice were subjected to LADL and the cardiac function was monitored with echocardiography. Beginning at the 4th week after the LADL, these mice were daily administrated with control solvent or HHQ16 for another 4 weeks. Results showed that compared with the *lnc9456*^f/f^ mice, the *lnc9456*^CKO^ mice had significantly higher LVEF and LVFS in both male (Fig. 7f, g) and female littermates (Supplementary Fig. 6g). They also displayed smaller overall heart size (Fig. 7h), lower HW/BSA ratio (Fig. 7i), smaller single myocyte size (Fig. 7j), lower expression of hypertrophy biomarkers ANP, BNP and β-MHC (Supplementary Fig. 6e), and less collagen deposition (Supplementary Fig. 6f) after 8 weeks of LADL, indicating that cardiomyocytes-specific knockout of *lnc9456* could protect cardiac function and prevent the development of the LADL-induced maladaptive remodeling and HF. Specifically, HHQ16 exhibited its inhibitory effects on cardiac hypertrophy and HF only in the *lnc9456*^f/f^ mice but not in the *lnc9456*^CKO^ mice (Fig. 7f–j, and Supplementary Fig. 6e–g), identifying *lnc9456* as the specific target for HHQ16 to reverse hypertrophy and HF.

Cardiomyocyte-specific deletion of *lnc9456* also abolished the inhibitory role of HHQ16 in fibrosis, suggesting that the effect of HHQ16 and *lnc9456* on fibrosis is dependent on their direct and primary effect on cardiomyocyte hypertrophy. As a matter of fact, it has been widely recognized that pathological cardiac hypertrophy is usually accompanied by cardiac fibrosis,^36,37^ and the improvement of cardiac hypertrophy would consequently result in the improvement of fibrosis,^15,38,39^ which is achieved possibly by the secondary changes of geometry shape, LV wall stress, loading condition and neurohormonal activation.^6,7^

The role of G3BP2 in the protection of HHQ16 against myocardial hypertrophy and HF was also evaluated (Supplementary Fig. 7). In both in vivo animal models of *lnc9456* overexpression- or LADL- induced cardiac hypertrophy (Supplementary Fig. 7a, b) and in vitro cell models of ISO- or ISO + PE- induced cardiomyocyte hypertrophy (Supplementary Fig. 7c, d), HHQ16 consistently caused a *lnc9456*-depedent reduction of G3BP2 in parallel with effective inhibition of hypertrophy. Furthermore, knockdown of G3BP2 eliminated the inhibitory effects of HHQ16 on hypertrophic markers (Supplementary Fig. 7e, f). These results further confirmed the critical role of *lnc9456*-depedent G3BP2/NF-κB pathway in the action of HHQ16 on myocardial hypertrophy and HF.

### Human ortholog *lnc4012* is a bona fide target for HHQ16

**Fig. 8:**
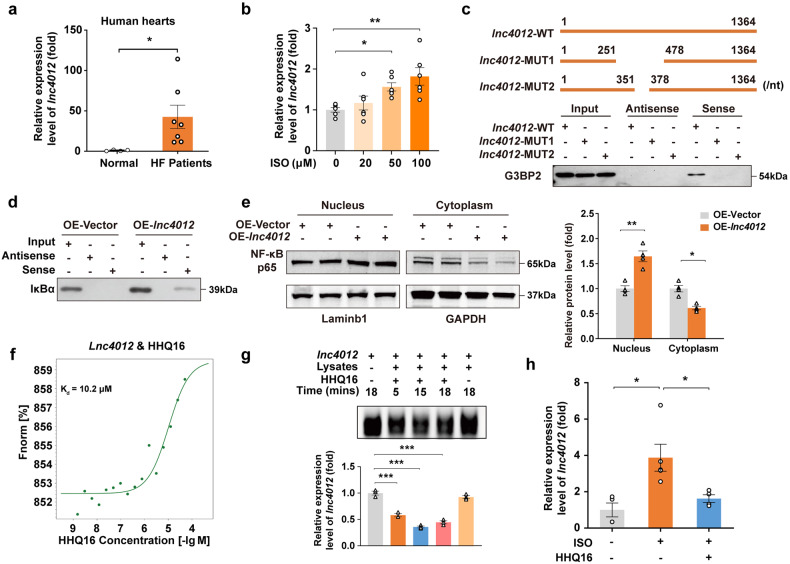
Human ortholog *lnc4012* is a bona fide target for HHQ16. **a** qRT-PCR detection for *lnc4012* of myocardial tissues derived from human normal or failing heart (*n* = 4–7). **b** qRT-PCR detection for *lnc4012* of AC16 human cardiomyocytes treated with vehicle or ISO (20, 50, 100 μM) for 24 h (*n* = 6). **c** Schematic diagram of full length *lnc4012* (*lnc4012*-WT), *lnc4012* truncated mutant1 (*lnc4012*-MUT1, Δ251-478 bp), and *lnc4012* truncated mutant2 (*lnc4012*-MUT2, Δ351-378 bp) (upper). RNA pull-down and western blotting detection for the binding of *lnc4012* to G3BP2 in AC16 human cardiomyocytes (lower) transfected with *lnc4012*-WT, *lnc4012*-MUT1 or *lnc4012*-MUT2. **d** RNA pull-down and western blotting detection for the binding of *lnc4012* to IκBα in AC16 human cardiomyocytes transfected with control plasmid (OE-NC) or *lnc4012* overexpression plasmid (OE-*lnc4012*) for 48 h. **e** Representative western blotting (left) and its quantification (right) for the expression of NF-κB p65 in nucleus or cytoplasm of AC16 human cardiomyocytes treated as mentioned in **d** (*n* = 4). **f** MST detection for the binding affinity of HHQ16 to *lnc4012*. *K*_d_ value was automatically by the curve fitting. **g** Representative agarose gel electrophoresis image (upper) and its quantification (lower) of in vitro-transcribed *lnc4012* incubated with vehicle or HHQ16 (100 nM) for the time indicated in the absence or presence of AC16 human cardiomyocyte lysates (*n* = 3). **h** qRT-PCR detection for *lnc4012* in Human Stem Cell Induced Differentiated Cardiomyocytes (HiPSC-CMs) treated with vehicle or ISO (50 μM) in the absence or presence of HHQ16 (100 nM) for 24 h (*n* = 4–5). Data are presented as the means ± SEM. *P < 0.05, **P < 0.01, ***P < 0.001 by Student’s *t* test (**a**), one-way ANOVA with Dunnett’s multiple comparisons test (**b**), two-way ANOVA with Sidak’s multiple comparisons test (**e**) and one-way ANOVA with Tukey’s multiple comparisons test (**g**, **h**)

Ortholog of *lnc9456* was further identified in human heart. Based on conserved genomic localization and its close distance to the human *EGR2* gene, *LOC107984012* (*lnc4012*) is the only gene located upstream of *EGR2* gene on Chromosome 10 and encodes a new 1064-nucleotide *lncRNA* with currently unknown function (Supplementary Fig. 8a). Strikingly, when compared to the hearts of healthy donors, expression of *lnc4012* was significantly upregulated by 36.06 ± 11.35-fold in the failing hearts from patients with dilated cardiomyopathy, a disease characterized by the eccentric hypertrophy^2^ (Fig. 8a), suggesting a close relationship of *lnc4012* upregulation with myocardial hypertrophy and HF.

To further define the function of this newly discovered *lnc4012* in the heart, the cell line of human cardiomyocyte, AC16 cells, were treated in vitro with ISO. As shown in Fig. 8b and Supplementary Fig. 8b, ISO not only stimulated the expression of the hypertrophy biomarkers ANP, BNP, and β-MHC but also increased the level of *lnc4012* in a dose-dependent manner, indicating that upregulation of *lnc4012* is indeed closely correlated with the increased hypertrophic gene transcription and the development of myocardial hypertrophy. Furthermore, overexpression of *lnc4012* in AC16 cells (Supplementary Fig. 8c) alone caused a significant increase in hypertrophy biomarkers in the absence of ISO (Supplementary Fig. 8d) while specific knockdown of *lnc4012* in these cells with smart silencer RNA (ssRNA, Supplementary Fig. 8e) suppressed the ISO-induced elevation of these markers (Supplementary Fig. 8f). These results indicated that an upregulation of *lnc4012*, same as the murine *lnc9456*, is a new pathogenic mechanism for the cardiomyocyte hypertrophy and plays a key role in promoting pathological hypertrophy and its progression to HF.

RNA pull-down assays were further performed in vitro with biotinylated *lnc4012* probe to explore its downstream mechanisms. The binding complexes generated by sense-*lnc4012* pull-down identified 1655 proteins by mass spectrometry. GO and KEGG enrichment analyses of these proteins yielded results highly similar to those of 2113 murine *lnc9456*-binding proteins with 12 identical items each in Top 20 (Supplementary Fig. 9a, b). GO analysis of RNA pull-down assay for *lnc4012* was also enriched in Poly(A) RNA binding, RNA binding and nucleotide binding (Supplementary Fig. 9a). Like *lnc9456* (Fig. 5f), the biotinylated human *lnc4012* sense probe (Sense-*lnc4012*) was able to capture more endogenous G3BP2 in the eluate of *lnc4012*-overexpressing cardiomyocytes (Fig. 8c). Furthermore, we interrogated the binding pattern of *lnc4012* to G3BP2 by catRAPID and identified a fragment, 351–378 bp of the transcript, as essential for binding (Supplementary Fig. 9c). Two mutant truncations of this fragment (251–478 bp and 351–378 bp) abolished the binding of *lnc4012* to G3BP2 protein (Fig. 8c). Compared with the control (OE-Vector), IκBα was detected only in the *4012*-overexpressing eluent (Fig. 8d). Overexpression of *lnc4012* also promoted translocation of NF-κB p65 from the cytoplasm to the nucleus (Fig. 8e). These results laterally corroborate the consistency of the molecular mechanism for murine *lnc9456* and human *lnc4012*.

Furthermore, HHQ16 had a higher affinity to *lnc4012* (*K*_d_ = 10.2 μM, Fig. 8f) than its murine ortholog *lnc9456* (*K*_d_ = 15.3 μM, Fig. 6f), and promoted the degradation of in vitro-transcribed *lnc4012* in a time-dependent manner (Fig. 8g). In the human induced pluripotent stem cell-derived cardiomyocytes (iPSC-CMs), HHQ16 significantly decreased ISO-induced expression of *lnc4012* (Fig. 8h), supporting the notion that human ortholog *lnc4012* is a bona fide target for HHQ16 to reverse pathological hypertrophy and HF.

## Discussion

In this study, we discovered that a new small molecule HHQ16, a derivative of *astragaloside IV*, reversed infarction-induced adverse remodeling and HF via directly regressing existing cardiac hypertrophy. The basis for the function of this small molecule was a newly identified transcript, *lnc9456* and its human ortholog *lnc4012*, which are responsible for the development of pathological hypertrophy and HF. HHQ16 specifically inhibited the hypertrophic effects of pathologically increased *lnc4012/lnc9456* by antagonizing their interactions with G3BP2 and blocking the NF-κB signaling pathway in the cardiomyocytes. These experimental results revealed a novel mechanism for the regression of infarction-induced hypertrophy and HF through targeted degradation of *lncRNA4012/9456* by HHQ16.

Post-infarction remodeling involves the remote non-infarcted myocardium and is defined by global LV dilatation and eccentric hypertrophy, in parallel with myocardial fibrosis. Progression rate from compensated cardiac hypertrophy to LV dysfunction is approximately 25% per 3 years after MI.^40^ Although initially viewed as a compensatory response, accumulating evidence suggests that this hypertrophy is not adaptive, but rather reflects the activation of maladaptive cellular processes that promote HF progression.^4,22,41^ Clinical studies have demonstrated the capacity of the heart to reverse the failing phenotype with the use of drug or surgical therapies in post-MI HF.^7–9^ Importantly, the observation that reverse remodeling and regress hypertrophy is associated with markedly improved myocyte and chamber contractility.^6,7^ Therefore, targeting pathological hypertrophy is emerging as a promising therapy for HF, a therapeutic focus away from neurohormonal systems to the heart muscle itself.^41,42^ HHQ16 may serve as such a small molecule compound. It is a novel optimized derivative of *astragaloside IV*, which displays an attractive in vivo activity of remodeling reversibility in mice with a failing heart caused by LAD ligation. Its best effect in improving cardiac function at 10 mg/kg is equivalent to Enalapril at 2 mg/kg and was even superior to LCZ696 at 100 mg/kg. Strikingly, HHQ16 showed potent activity in directly reversing cardiac hypertrophy both in vivo and in vitro, significantly ameliorating the cardiomyocyte abnormalities caused by multiple pathological stimuli, suggesting that HHQ16 is an attractive candidate drug in the treatment of MI-induced adverse remodeling and HF.

Advances in sequencing technologies of transcriptome have provided platforms for exploring new insights into the mechanisms for reverse remodeling, especially by non-coding RNAs. *Mhrt*,^35^ *Chaer*,^43^ *Chast*,^44^ *Meg3*^45^ and *H-19*^46^ have been found to be involved in the pathological processes of pressure-overload caused LV hypertrophy in animal models. These findings opened up a promising field for HF drug development to pharmacologically target at *lncRNAs* as a therapeutic strategy.^47^ However, it remains largely unknown how these *lncRNAs* dynamically regulate and control the hypertrophic remodeling and progression process and their significance in human cardiomyopathy and HF. Our current study defined human *lnc4012* and its murine ortholog *lnc9456* as a new key mechanism that promotes the cardiac hypertrophy and HF progression in the face of pathological stresses. The human *lnc4012* in the hypertrophied failing hearts of DCM patients and its murine ortholog *lnc9456* in the hypertrophied failing hearts of LADL mice are both significantly upregulated, while they are minimally expressed in the normal heart. Importantly, cardiac myocyte-specific over-expression of *lnc4012*/*lnc9456* alone can cause myocardial hypertrophy and dysfunction while specific knockdown or knockout of *lnc4012*/*lnc9456* can prevent cardiac hypertrophy caused by ISO, Ang II, ISO + PE, and ischemic stresses. HHQ16 specifically inhibited the hypertrophic effects of pathologically increased *lnc4012*/*lnc9456* by antagonizing their interaction with G3BP2 and blocking the NF-κB signaling pathway in the myocytes and reversed LV remodeling and HF. For the first time in the literature, we provide compelling evidence that targeted degrading the pathologically upregulated *lncRNAs* may provide a novel strategy to develop RNA-targeting drugs.

The RNA binding protein family G3BPs regulate gene expression by controlling RNA stability and translation.^48^ The two G3BP members, G3BP1 and G3BP2, have different functions.^49^ While G3BP1 was extensively studied and known as a multi-functional protein in the assembly and dynamics of stress granules as well as functions related to RNA metabolism, much less is known about the function of G3BP2. A previous study found that G3BP2 might participate in cardiac hypertrophy caused by ISO-stimulation through manipulating the nucleoplasmic distribution of IκBα.^33,34^ The other study found that IκBα and IκBα/NF-κB complexes are retained in the cytoplasm through interaction with G3BP2.^50^ It has been well known that the subcellular localization of IκBα is critical for the regulation of NF-κB transcriptional activity.^51,52^ Under pathophysiological conditions such as ISO-stimulation, the increased G3BP2 may anchor IκBα in the cytoplasm and promote nuclear aggregation of the NF-κB subunit p65, which in turn activates the NF-κB pathway and triggers a series of hypertrophic, inflammatory, and other related transcriptional responses that ultimately lead to related events such as cardiac remodeling and dysfunction. Our current study showed that high concentration of *lnc4012/9456* interacts with G3BP2 and increases its association with IκBα. This explains why overexpression of *lnc4012/9456* can cause cardiac hypertrophy and inhibition of *lnc4012/9456* can inhibit cardiac hypertrophy both in vitro and in vivo.

Pathological myocardial hypertrophy (PMH) is a dynamic maladaptive response of the heart to a variety of etiological stimuli, such as hypertension, MI, valvular heart disease, diabetes, and many others.^2^ Interestingly, Ang II or ISO-induced myocytes hypertrophy was abolished when *lnc4012/lnc9456* was knocked down, indicating that pathological upregulation of *lnc4012/lnc9456* might represent the downstream mediator for hypertrophic responses to neurohormonal activation. Although the causes of HF vary, the derangement of neurohormonal activation is a basic pathophysiology to promote the progression of HF.^53^ Therefore, selective inhibition of *lnc4012/lnc9456* would represent a potentially effective approach toward reversing myocardial remodeling and the subsequent development to HF. Clinical studies have found many factors, including age, sex, BMI and disease etiology, affect the reversibility of remodeling and hypertrophy. The therapeutic efficacy of a drug needs to be interpreted in the context of the underlying pathophysiology of PMH.^8,41^ Thus, the activity of HHQ16 in the model of MI-induced PMH may not necessarily be applicable for remodeling caused by other etiologies, such as pressure overload-induced PMH, but is worthy of being further explored in the future.

In conclusion, we have comprehensively characterized a newly discovered human *lncRNA* (*lnc4012*) and its murine ortholog (*lnc9456*) and provided novel mechanistic insights into the roles of *lnc4012*/*lnc9456* in the development of MI-induced hypertrophy and HF. We also provided compelling evidence that *lnc4012*/*lnc9456* is a new bona fide target for a new small molecule HHQ16 to effectively reverse infarction-induced hypertrophy and HF by specific binding to *lnc4012*/*lnc9456*, causing its degradation, and antagonizing its action on the G3BP2/NF-κB signaling in cardiomyocyte. Targeted degradation of pathological increased *lnc4012/lnc9456* by small molecules might serve as a novel promising strategy to regress, possibly not merely MI-induced, cardiac hypertrophy and HF.

## Materials and methods

### Human heart samples

The cDNAs of human heart samples, from patients diagnosed with DCM and end-stage HF who underwent heart transplantation (*n* = 7) and age-matched donors without any heart disease (*n* = 4), were kindly gifted by Professor Peiqing Liu and Jing Lu (School of Pharmaceutical Sciences, Sun Yat-sen University, Guangzhou, China). The human heart samples were obtained from the Second Department of Cardiac Surgery, First Affiliated Hospital of Sun Yat-sen University (Guangzhou, China) and the ethic approval number was No. [2017]157. The study conformed to the principles outlined in the Declaration of Helsinki. Informed consent was obtained from the families of the subjects.^54^ The expression of *lnc4012* in these samples was detected by qRT-PCR.

### The optimization, synthesis and physical-chemical properties of HHQ16

The efficacy of a series of *Astragaloside IV* derivatives were firstly evaluated as mentioned in Fig. 1b. Briefly, chronic HF model was established at 4 weeks post-LADL in mice. These derivatives were daily intragastric administrated for consecutive 4 weeks, then cardiac function was detected to find lead compounds. For these lead compounds, further druggable studies were conducted, including structure optimization, preparation process optimization, in vitro and in vivo pharmacokinetics bioavailability, and early safety. Based on these data, HHQ16 was finally chosen as the best appropriate candidate molecule.

The synthesis of HHQ16 proceeds through seven sequential steps. *Astragaloside IV* is employed as the starting material, and the synthesis involves hydrolysis, selective hydroxyl protection, acetylation, hydroxyl deprotection, oxidation, fluorination, and deacetylation protection, comprising a total of seven distinct reactions. HHQ16 exhibits a white crystalline solid appearance. Its chemical formula is C_30_H_48_F_204_ with a molecular weight of 510.71 g/mol. It possesses a polar surface area (PSA) of 69.92 and a Log P (partition coefficient) value of 6.78.

### Animals

Male C57BL/6J mice (20 ± 2 g) were purchased from Sino-British SIPPR/BK Lab Animal Ltd (China). Cardiomyocyte-specific *lnc9456-*deficient (Myh6^Cre^ × *lnc9456*^flox/flox^, *lnc9456*^CKO^) mice were generated by Shanghai Model Organisms Center, Inc (Shanghai, China) using CRISPR/Cas9 system in C57BL/6J mouse background. The *lnc9456* donor vector containing flox sites flanking exons 1 of *lnc9456* gene with two gRNAs (5’-CCCCGCGTATCTCACCAATTCGG-3’; 5’-AAGGAGAATGTATTGCCAATTGG-3’) and Cas9 mRNA was microinjected into C57BL/6J fertilized eggs. F0 generation mice positive for homologous recombination were identified by long PCR. The primers used for genotyping were P1: 5’-GGTGCCAGGCTATCAAGACA-3’ and P2: 5’-TCCGCTCCTGCTTGCTTATC-3’ for the 5’ homology arm recombination, and P3: 5’-GCCAAGTTTTCCACATGGGT-3’ and P4: 5’-CACTATTCACGAACCGCGTG-3’ for the 3’ homology arm recombination. The positive founder mice were mated to wild-type mice to obtain flox heterozygous mice. The homozygous mice were crossed with Myh6-CreERT2 mice to generate inducible cardiomyocyte-specific knockout mice and control mice. To achieve specific knockout of the *lnc9456* gene, mice were daily intraperitoneal injection of 75 mg/kg of tamoxifen (Sigma, cat. no. 10540-29-1) for consecutive 5 days, and then experienced a 7-day waiting period before the beginning of experiments. Animal care and use for this study were performed respecting the National Institute of Heath’s Guide for the Care and Use of Laboratory Animals and approved by the Laboratory Animal Care and Use Committee of the Second Military Medical University, China.

### Cell lines and cultures

All cells were cultured in a 37 °C 5% CO_2_ humidified incubator. HL-1 mouse cardiomyocytes line and AC16 human cardiomyocytes line were purchased from Procell or Millipore and cultured in DMEM high glucose medium (Gibco, cat. no. 11995-065) containing 10% FBS (Gibco, cat. no. 10099-141C) and 1% Penicillin-Streptomycin (Gibco, cat. no. 15140-122). Primary cardiomyocytes were extracted using two-step method from 1–3 days neonatal mice as described previously^55^ and cultured in DMEM high glucose medium containing 15% FBS and 1% Penicillin-Streptomycin. Human Stem Cell Induced Differentiated Cardiomyocytes (HiPSC-CMs) were purchased from Help Stem Cell Innovations and cultured with NovoCell^TM^-Cardiomyocytes Kit (Help Stem Cell Innovations, cat. no. HELP4111).

### Left anterior descending ligation (LADL) model and echocardiography

Surgical anesthesia was achieved with the inhalation of 2.5% of isoflurane, and the heart was exposed by the left thoracotomy. Subsequently, ligation was performed by constructing a slipknot (7–0 silk) around the left anterior descending. In the sham-operated group, the suture was just passed around the coronary artery but not ligated. Four weeks after surgery, mice with chronic heart failure were screened by echocardiography. Briefly, transthoracic echocardiography was measured in anaesthetized mice using the position of supine or left lateral. Two-dimensional long axis images were recorded by using a MyLab twice high-resolution in vivo micro-imaging system (Esaote, Italy) with an SL3116 transducer. The left ventricle (LV) ejection fraction (EF) and fractional shortening (FS) were obtained by M-mode at the level of the papillary muscles.

### Histological analyses

The remote cardiac muscle tissues in the non-infarct region, 2–3 mm away from the ligation site, were harvested. Fresh heart tissues or HL-1 mouse cardiomyocytes were fixed in 4% paraformaldehyde. Heart tissues were further embedded in paraffin according to standard protocols and sectioned into 5-μm-thick slices, and histological evaluation was performed as described previously.^56,57^ Briefly, hematoxylin and eosin (HE), wheat germ agglutinin (WGA, Sigma-Aldrich, cat. no. L4895) and Masson’s trichrome stain were used to evaluate heart histological features, cell size and collagen deposition, respectively. Phalloidin stain was used to evaluate size of HL-1 cells. Immunofluorescence staining was used to assess cellular localization of NF-κB p65 in HL-1 cells by using NF-κB p65 antibody (Cell Signaling, cat. no. D14E12). Photos were taken under a digital slice scanner (Pannoramic MIDI, 3DHistech). The relative size of cardiomyocytes was quantified by a densitometric analysis using ImageJ software (National Institutes of Health). Specifically, two fields of view were obtained randomly for each sample. For each field, the sizes of 12 cells were obtained, and the average value from total 24 cells was calculated as the final cell size. The collagen area was quantified by using the Image Pro Plus software (Media Cybernetics, Inc) and calculated as the percentage of collagen area to whole tissue area.

### Electron microscopy

Electron microscopy was performed as described previously.^56^ Mouse heart tissue was cut into small pieces (1–2 mm^3^) and fixed in 2.5% of glutaraldehyde. These pieces are sliced by Leica EM UC7 (Leica) and photographed using Hitachi transmission electron microscope (HT7700, 120kv, Hitachi). The mitochondria number per image was counted.

### Western blotting

Protein extraction and western blotting were performed as previously reported.^56^ Briefly, total proteins were extracted from lysis buffer containing protease inhibitor cocktail (Kangchen, cat. no. KC-415), and nuclear or cytoplasmic proteins were extracted using the Minute Cytoplasmic and Nuclear Fractionation kit (Invent, cat. no. SC-003). Proteins (30 μg) were subjected to SDS-PAGE using polyacrylamide gels. Immunoblotting was performed using specific antibodies (ANP, Beyotime, cat. no. AF7608; BNP, Abcam, cat. no. ab236101; β-MHC, Abcam, cat. no. ab172967; NF-κB p65, Cell Signaling, cat. no. D14E12; IκBα, Cell Signaling, cat. no. 9242; G3BP2, Affinity Biosciences, cat. no. DF4387, TUBULIN, Beyotime, cat. no.AF1216; GAPDH, Beyotime, cat. no. AF1186 and Lamin B1, Proteintech, cat. no.12987-1-AP) to evaluate the expression of proteins. The image was acquired using the Odyssey infrared fluorescence imaging system (Li-Cor, Lincoln, NE). Band intensity was quantified by a densitometric analysis using ImageJ software (National Institutes of Health).

### Quantitative real-time PCR (qRT-PCR)

Total RNA was isolated by Trizol reagent (Takara, cat. no. 9109) and reverse-transcribed to cDNA using PrimeScript^TM^ RT Master Mix (Takara, cat. no. RR036A). Real-time quantitative PCR analysis was performed using FastStart Universal SYBR Green Master kits (Roche, cat. no. 04913914001). Gene expression levels were normalized to the control gene *Gapdh*. The primer sequences for the genes were: *Anp*, 5’-GCTTCCAGGCCATATTGGAG (forward), 5’-GGGGGCATGACCTCATCTT-3’ (reverse); *Bnp*, 5’-GAGGTCACTCCTATCCTCTGG-3’ (forward), 5’-GCCATTTCCTCCGACTTTTCTC-3’ (reverse); *β-Mhc*, 5’-ATGTGCCGGACCTTGGAA-3’ (forward), 5’-CCTCGGGTTAGCTGAGAGATCA-3’ (reverse); *Gapdh*, 5’-TGGATTTGGACGCATTGGTC-3’ (forward), 5’-TTTGCACTGGTACGTGTTGAT-3’ (reverse); *lnc9456*, 5’-AGCATCACTACGGCAGCTTACAAC-3’ (forward), 5’-AGGTTCACAGGACTCTGACGACTC-3’ (reverse); *lnc4012*, 5’-GCTTGCTTCCTACTCTGCCATAAGG-3’ (forward), 5’-GGCACTGGTGGAACTGGATGAC-3’ (reverse).

### RNA sequencing and analysis

Total RNA was extracted using the Trizol reagent according to the manufacturer’s protocol. RNA integrity was assessed using the Agilent 2100 Bioanalyzer (Agilent Technologies). Libraries were established using TruSeq Stranded Total RNA with Ribo-Zero Gold (Illumina), and sequenced pair end on the Hiseq X-ten platform (Illumina). Raw data of fastq format were filtered with the Trimmomatic software, and the clean reads were mapped to mouse genome (GRCm39/mm39) using HISAT2. For mRNAs, FPKM and the read counts of each gene were obtained for downstream analysis. Differential expression analysis was performed using the DESeq (2012) R package. |log2 (Fold Change)| > 1 and *p* value < 0.05 was set as the threshold for significantly differential expression. For *lncRNAs*, the transcriptome from each dataset was assembled independently, and all transcriptomes were pooled and merged to generate a final transcriptome. |Fold Change| > 5 and *p* value < 0.05 was set as the threshold for significantly differential expression.

### RNA pull-down assay

Pierce™ Magnetic RNA-Protein Pull-Down Kit (Thermo Fisher, cat. no. 20164) provides reagents to efficiently enrich RNA Binding Proteins. Sense or antisense of *lnc9456* or *lnc4012* were in vitro transcription using the T7 RNA transcription system (Large Scale RNA ProductionSystem-T7, Promega, cat. no. P1300) and biotin-labeled with the Pierce™ RNA 3’ End Desthiobiotinylation Kit (Thermo Scientific, cat. no. 20163). The primer sequences were: *lnc9456*-sense: 5’-TAATACGACTCACTATAGGGGTGACACATCTGGAGATTTTCC-3’ (forward), 5’-AGTCAGAGGATAACTTGGGGG-3’ (reverse); *lnc9456*-antisense: 5’-GTGACACATCTGGAGATTTTCC-3’ (forward), 5’-TAATACGACTCACTATAGGGAGTCAGAGGATAACTTGGGGG-3’ (reverse); *lnc4012*-sense: 5’-TAATACGACTCACTATAGGGCAGGCCAAGCTTGCTTCCTAC-3’ (forward), 5’-TCAAGCCATGAGTCAGCCTAA-3’ (reverse); *lnc4012*-antisense: 5’-CAGGCCAAGCTTGCTTCCTAC-3’ (forward), TAATACGACTCACTATAGGGTCAAGCCATGAGTCAGCCTAA-3’ (reverse). RNA was then purified with QIAquick PCR Purification Kit (Qiagen, cat. no. 28106). 30 μg of whole-cell protein lysates were incubated with purified biotinylated transcripts for 1 h at 4 °C with rotation, then they were eluted for mass spectrometry identification. RNA pull-down and analysis were performed by Shanghai Dianxi Biotechnology Co., Ltd.

### Co-immunoprecipitation (Co-IP)

Co-IP was performed with Pierce Crosslink Magnetic IP/Co-IP Kit (Thermo Fisher, cat. no. 88805). Briefly, 7 μg of IκBα antibody was bound to Pierce Protein A/G Magnetic Beads for 15 min and crosslinked to beads with disuccinimidyl suberate (DSS) for 30 min. Cell lysates with 1500 μg total protein were incubated overnight with antibody-crosslinked beads at 4 °C with rotation. Bound antigen was eluted, and the immunoprecipitates were subjected to SDS-PAGE and standard western blotting procedures.

### Plasmid construction and transfection

The overexpression plasmid of *lnc9456* or *lnc4012* and their negative control vectors were constructed by GenePharma. The full length of *lnc9456* or *lnc4012* were inserted into pGCMV/MCS/Neo eukaryotic expression vector with CMV promoter, and plasmids were transfected by GP-transfect-Mate (GenePharma). The adenovirus harboring *lnc9456* (AdV-*lnc9456*) was also constructed by GenePharma, and transfected by polybrene (Sigma-Aldrich). The adeno-associated virus harboring *lnc9456* (AAV-*lnc9456*) was constructed using cTNT myocardial specific promoter by Hanbio Biotechnology. Mice were intramyocardially injected with 50 μL of AAV-*lnc9456* or the corresponding control.

### RNA interference

The *lncRNA* smart silencer is a mixture of three anti-sense oligonucleotides (ASO) and three small interference RNAs (siRNAs), which could effectively knock down both nuclear and cytoplasmic *lncRNAs*.^58^ *LncRNA* smart silencer for *lnc9456* or *lnc4012* and small interference RNAs (siRNAs) for *G3bp2* were purchased from Ribobio. They were transfected into cells at optimal final concentration of 50 nM by using the riboFECT™ CP transfection reagent (Ribobio).

### Rapid amplification of cDNA ends (RACE)

Total RNA was isolated using TRIzol Plus RNA Purification Kit (Invitrogen, cat. no. 12183-555), and 5’ RACE and 3’ RACE was performed using GeneRacer™ Kit (Invitrogen, cat. no. L1500-01) according to the manufacturer’s instructions. Primers used for RACE and nested PCR are: 5’-GGCTATGGCTGTGACTCGGCTTTC-3’ (5’ RACE *lnc9456*-1), 5’-GTAAAACTGCAGAAGCTGTTTGCTTAAGAC-3’ (5’ RACE *lnc9456*-2), 5’-GGTTAGACCCTGGGTCTTGGTAGGTAGCT-3’ (3’ RACE *lnc9456*-1), 5’-GCTCCTGACTTATCGAGAGAGCCTGTCTCA-3’ (3’ RACE *lnc9456*-2). RACE products were separated by agarose gel electrophoresis, purified from the gel, and analyzed by DNA sequencing.

### In vivo imaging of mice

Mice were anesthetized and injected intraperitoneally with 150 mg/kg of D-luciferin potassium salt (Shanghai Liji, cat. no. ac19l012). Subsequently, the in vivo imaging of heart was captured by the Chemiluminescence Fluorescence Image Analysis System (PerkinElmer) at 10–20 min after injection.

### Bioinformatics analysis

The catRAPID omics module was used to predict the possible interacting proteins with *lnc9456*. The binding propensity of G3BP2 protein and *lncRNA lnc9456/lnc4012* was estimated via algorithms supplied by catRAPID, and the binding regions between them was predicted via the catRAPID fragments module.

### Microscale thermophoresis (MST)

The MST detection for the binding of *lnc9456/lnc4012* to HHQ16 was performed as described previously.^59^ Briefly, the 5’ Cy5-modified *lnc9456/lnc4012* was obtained using the T7 in vitro transcription system (Invitrogen). The Cy5-labeled *lnc9456/lnc4012* was diluted in TBST buffer to provide the optimal level of the fluorescent intensity. In total, 16 titration series of HHQ16 were prepared beginning at a concentration of 100 μM and mixed with labeled *lnc9456/lnc4012*. Subsequently, 5 μL of RNA solution was mixed with 5 μL of HHQ16 in different concentrations. After 5 min incubation at room temperature, all the samples were loaded into MST NT.115 (NanoTemper) standard glass capillaries and measurement were carried out using the MO. Control software. The equation for calculating *K*_d_ is $f\left(c\right)={Unbound}+\left({Bound}-{Unbound}\right)\times \frac{c+{c}_{{target}}+{K}_{d}-\sqrt{{\left(c+{c}_{{target}}+{K}_{d}\right)}^{2}-4c\,{c}_{{target}}}}{2\,{c}_{{target}}}$.

### RNA stability assay in vitro

#### Cell lysates preparation

HL-1 mouse cardiomyocytes or AC16 human cardiomyocytes lysates were prepared as previously described^60^ with some modifications. Briefly, cardiomyocytes were harvested by trypsinization, and washed twice in ice-cold PBS and once in low salt buffer (LSB: 50 mM Hepes-NaOH, pH7.5, 5 mM KCl, 1.5 mM MgCl_2_, 1 mM DTT). The pellet of cardiomyocytes was then resuspended in the LSB solution, lysed by sonication at 4 °C. To prepare whole-cell extracts, cellular debris was removed by centrifugation at 11,000 × *g* for 20 min. All cardiomyocyte lysates were aliquoted and stored at −80 °C.

#### The in vitro transcription of *lnc9456/4012*

The *lnc9456/4012* was prepared by in vitro transcription using in vitro transcription kit (Invitrogen, cat. no. AM1333). The PCR products which contain the T7 RNA polymerase promoter site had been used as templates for the in vitro transcription of *lnc9456/4012*. Primers were synthesized by GENWIZ. The *lnc9456* plasmid was amplified by PCR using 5’-GGTAATACGACTCACTATAGCGGTGACACATCTGGAGATTTTCCAC (forward) and 5’-TGCTTTCCATCCTTTTTCTTTATTAAAATTG (reverse). The *lnc4012* plasmid was amplified by PCR using 5’-GGTAATACGACTCACTATAGCGTGCTTACCAAGTTCTAAGGCCTTACTGAT (forward) and -5’- TCAAGCCATGAGTCAGCCTAACTCA (reverse). The *lnc9456****/****4012* was purified by VAHTS RNA clean beads following the in vitro transcription.

#### Degradation assay of *lnc9456/4012* by HHQ16 in cell lysates

The total reaction volume was 10 µL, were taken in each tube and comprised of: cardiomyocytes lysates, HHQ16 (DMSO)/DMSO, *lnc9456/4012*, and DNase/RNase-free water. Briefly, 1 µL of cardiomyocytes lysates were mixed with 1 µL of HHQ16/DMSO, and incubated with 2 µL of *lnc9456/4012*, then supplemented with 6 µL of DNase/RNase-free water. The final concentration of HHQ16 and *lnc9456/4012* added to the reaction were 100 nM and 0.15 µM, respectively. The reaction was incubated at 37 °C. *Lnc9456/4012* degradation products were mixed with 6 × DNA Loading buffer and separated on a 0.8% agarose gel. The gel was imaged with ChemiDoc XRS+ System (BIO-RAD, US).

### RNA stability assay in cells

The transcription of *lnc9456* was shut off by adding the transcription inhibitor actinomycin D (ActD) into the cell culture medium with the final concentration of 0.5 μg/mL, along with 100 nM of HHQ16 or corresponding vehicle. After 0, 1, 3, and 6 h, total RNA was respectively collected and extracted for qRT-PCR analysis. Normalize the Ct average of each time point to the Ct average value of *t* = 0 h to obtain ∆Ct value.

### Statistical analysis

Data are presented as mean ± SEM. One- or two-way ANOVA or Student’s *t* test was performed for multiple comparisons as necessary. The criterion of statistical significance was *P* < 0.05, and symbols indicate *P* values as follows: *P < 0.05; **P < 0.01; ***P < 0.001. Statistical analyses were conducted with GraphPad Prism 8 software.

### Supplementary information

Supplementary Materials

## Data Availability

The RNA sequencing data was deposited at the NCBI (BioProject ID: PRJNA882368). Proteomics mass spectrometry data of RNA pull down was deposited at ProteomeXchange (PXD036966 and PXD037001). Any additional information required for reanalysis is available upon request.
