# Supplementary material for: Astragaloside IV derivative HHQ16 ameliorates infarction-induced hypertrophy and heart failure through degradation of lncRNA4012/9456
Source: Signal Transduct Target Ther. 2023 Oct 19;8:414. doi: 10.1038/s41392-023-01660-9 (PMC10587311; doi:10.1038/s41392-023-01660-9)
Supplement: Supplementary file 1 — Supplementary Materials [file 41392_2023_1660_MOESM1_ESM.docx]

Supplementary Materials for

**Astragaloside IV derivative HHQ16 ameliorates infarction-induced hypertrophy and heart failure through degradation of *lncRNA4012/9456***

Jingjing Wan^1^, Zhen Zhang^1^, Chennan Wu^1^, Saisai Tian^1^, Yibei Zang^1^, Ge Jin^1^, Qingyan Sun^2^, Pin Wang^3^, Xin Luan^4^, Yili Yang ^5^, Xuelin Zhan^5, 6^, Lingyu Linda Ye^7^, Dayue Darrel Duan^7,8^, Xia Liu^1^, Weidong Zhang^1,9^

Correspondence: Weidong Zhang (wdzhangy@hotmail.com), Xia Liu ([lxflying@aliyun.com](mailto:lxflying@aliyun.com)), and Dayue Darrel Duan (dduan@swmu.edu.cn).

These authors contributed equally: Jingjing Wan, Zhen Zhang.

**This PDF file includes:**

Supplementary Fig. 1 to Fig. 9

Supplementary Table 1


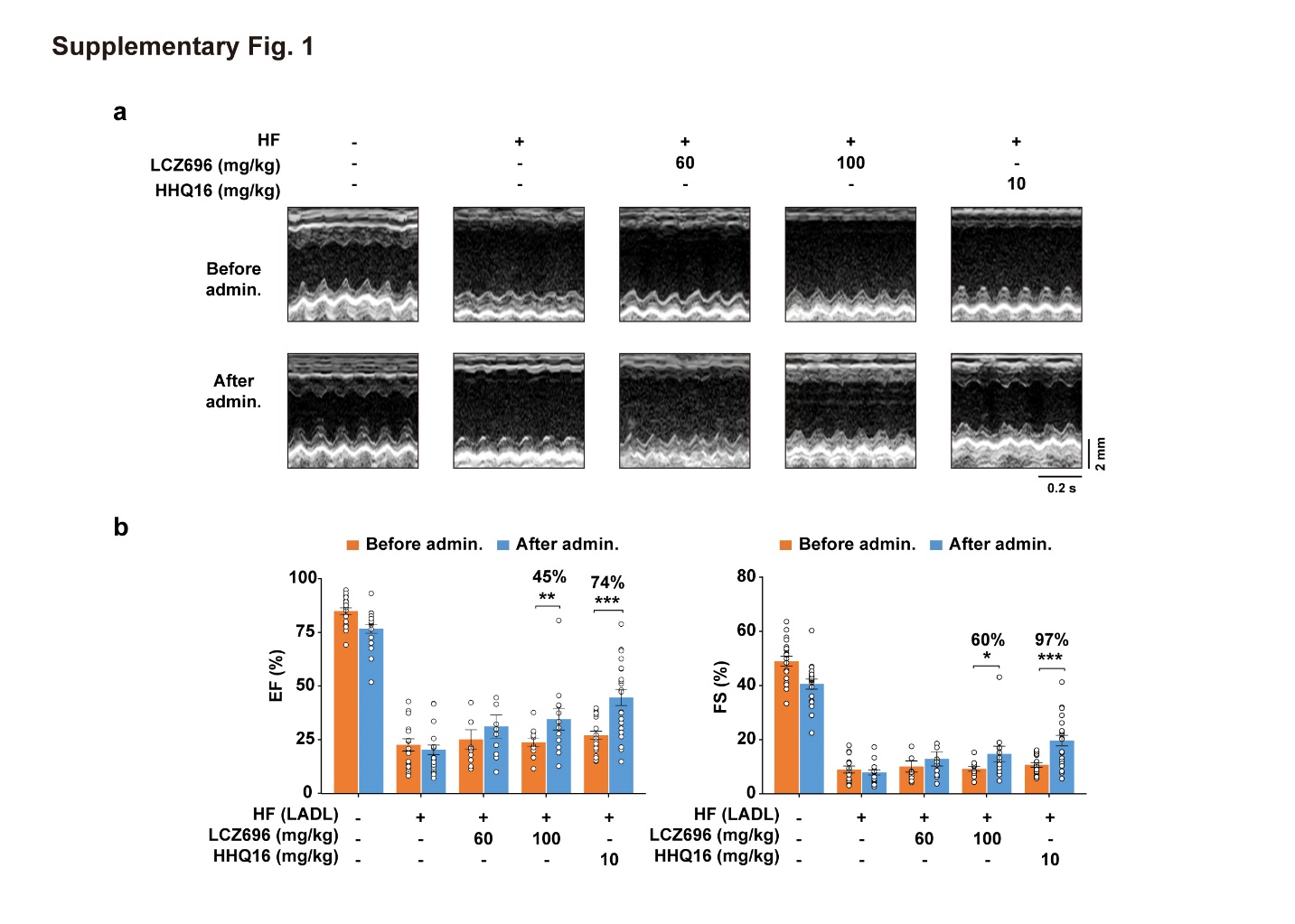


**Supplementary Fig. 1 HHQ16 significantly improves cardiac function in HF mice.**

**a, b** Representative M-mode images (**a**) and statistical analysis of EF (%) and FS (%) (**b**) in mice underwent sham or LADL surgery for 4 weeks, then treated by daily intragastric administration of vehicle, LCZ696, or HHQ16 at the doses of indicated for 4 weeks. The cardiac function was detected by echocardiography at 4 (before admin.) and 8 (after admin.) weeks post-LADL (n=10-22). Data are presented as the means ± SEM. *P<0.05, **P<0.01, ***P<0.001 by two-way ANOVA with Sidak’s multiple comparisons test (**b**).


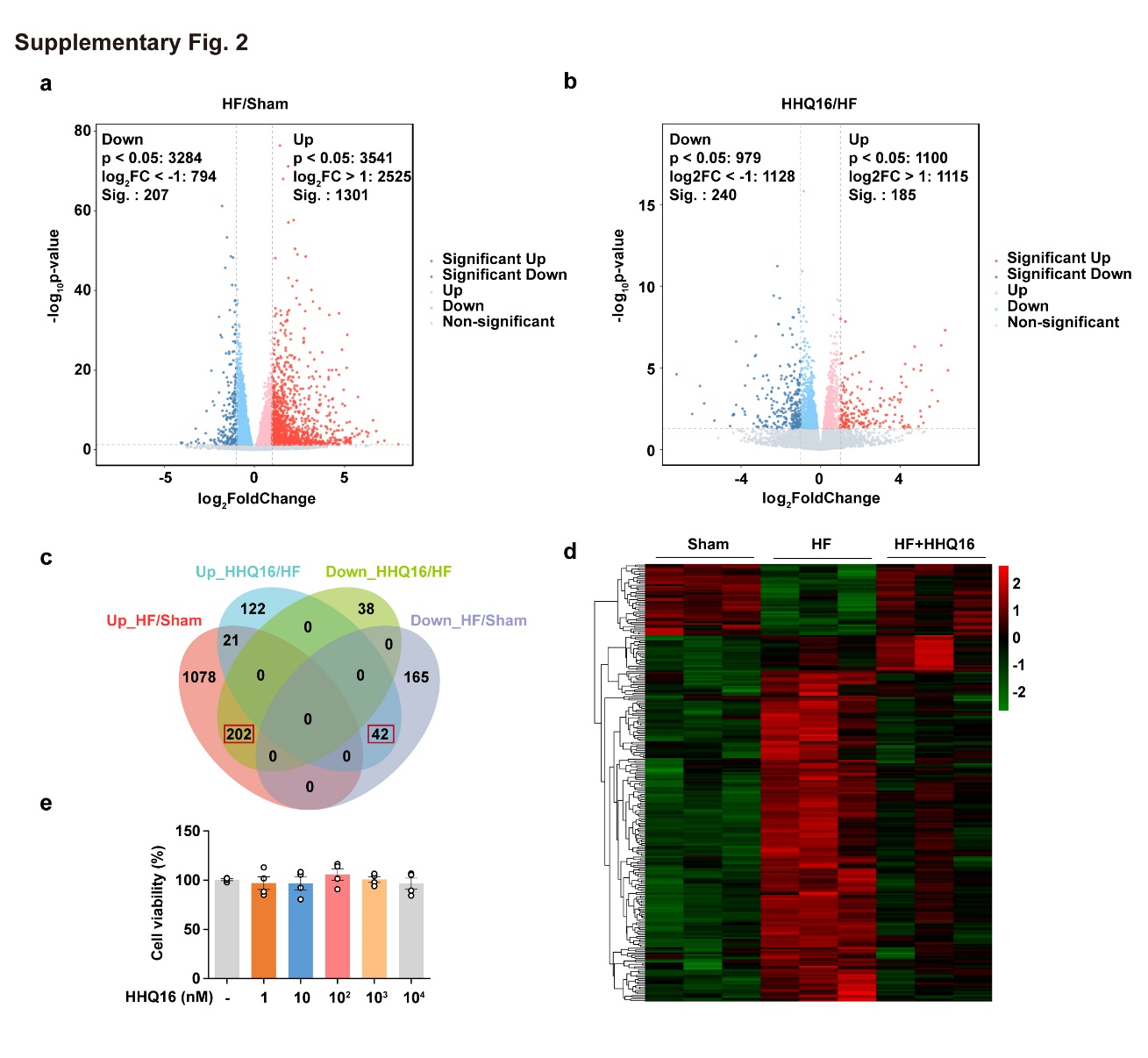


**Supplementary Fig. 2 Analysis of differentially expressed genes (mRNAs) in HF mice treated with HHQ16.**

**a, b** Volcano plot shows the DEGs (differentially expressed genes) discovery by using the p <0.05 and |foldchange (FC)| ≥ 2 as the threshold of significant difference. Colored points represent differentially expressed mRNAs with |foldchange (FC)| ≥ 2 between Sham and HF group (**a**), or HF+HHQ16 and HF group (**b**), red represent upregulated genes and blue represent downregulated genes. **c** Venn diagram of mRNAs with different regulatory directions. **d** Heatmap shows the upregulated (red) and downregulated (green) expression of genes in Sham, HF and HF+HHQ16 group. **e** The CCK8 assay of neonatal mouse primary cardiomyocytes treated with vehicle or HHQ16 at the indicated doses for 24h (n=4). Data are presented as the means ± SEM. Statistical analysis was conducted by one-way ANOVA with Tukey’s multiple comparisons test.


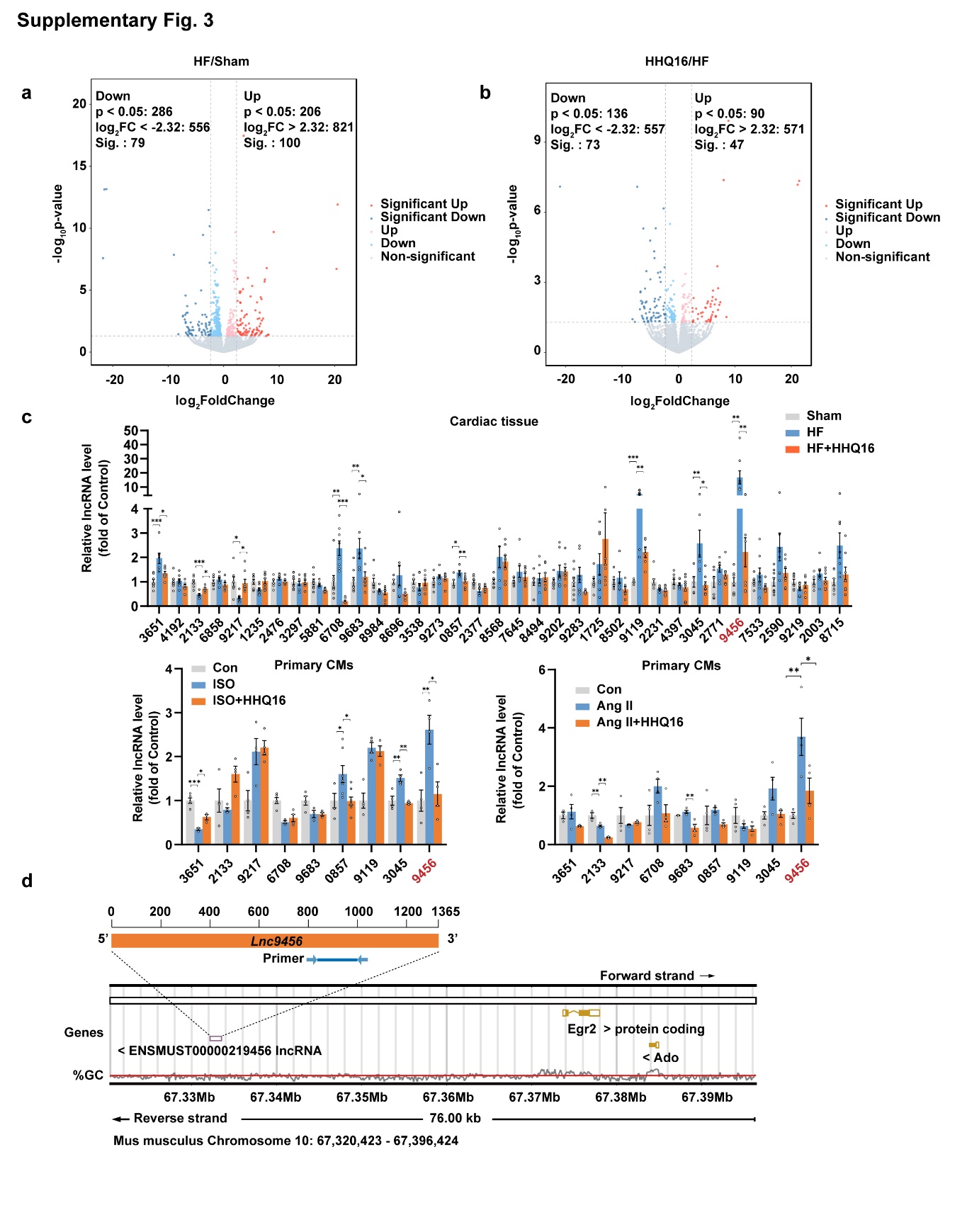


**Supplementary Fig. 3 Analysis of differentially expressed transcripts (*lncRNAs*) in HF mice treated with HHQ16.**

**a, b** Volcano plot shows the DETs (differentially expressed transcripts) discovery by using the p <0.05 and |foldchange (FC)| ≥ 5 as the threshold of significant difference. Colored points represent differentially expressed *lncRNAs* with |foldchange (FC)| ≥ 5 between Sham and HF group (**a**), or HF+HHQ16 and HF group (**b**), red represent upregulated genes and blue represent downregulated genes. **c** The finding procedure of *lnc9456*. Firstly, qRT-PCR detection of 35 candidate transcripts mentioned in Fig. 3**a** was performed using the myocardial tissues mentioned in Fig. 1**e** (upper), identifying 9 transcripts as significantly affected ones by HHQ16. Subsequently, the 9 transcripts were further detected by qRT-PCR in hypertrophic primary mouse cardiomyocytes treated with or without HHQ16 (100 nM), identifying *lnc9456* as the only significantly affected transcript by HHQ16 in both ISO (lower, left)- and Ang Ⅱ (lower, right)- induced hypertrophic models. **d** Genomic locus of the *lnc9456* and its adjacency to the *Egr2* gene from the Ensemble database. The schematic diagram indicates the binding regions of qRT-PCR primers on *lnc9456* gene. Data are presented as the means ± SEM. *P<0.05, **P<0.01, ***P<0.001 by one-way ANOVA with Tukey’s multiple comparisons test (**c**).


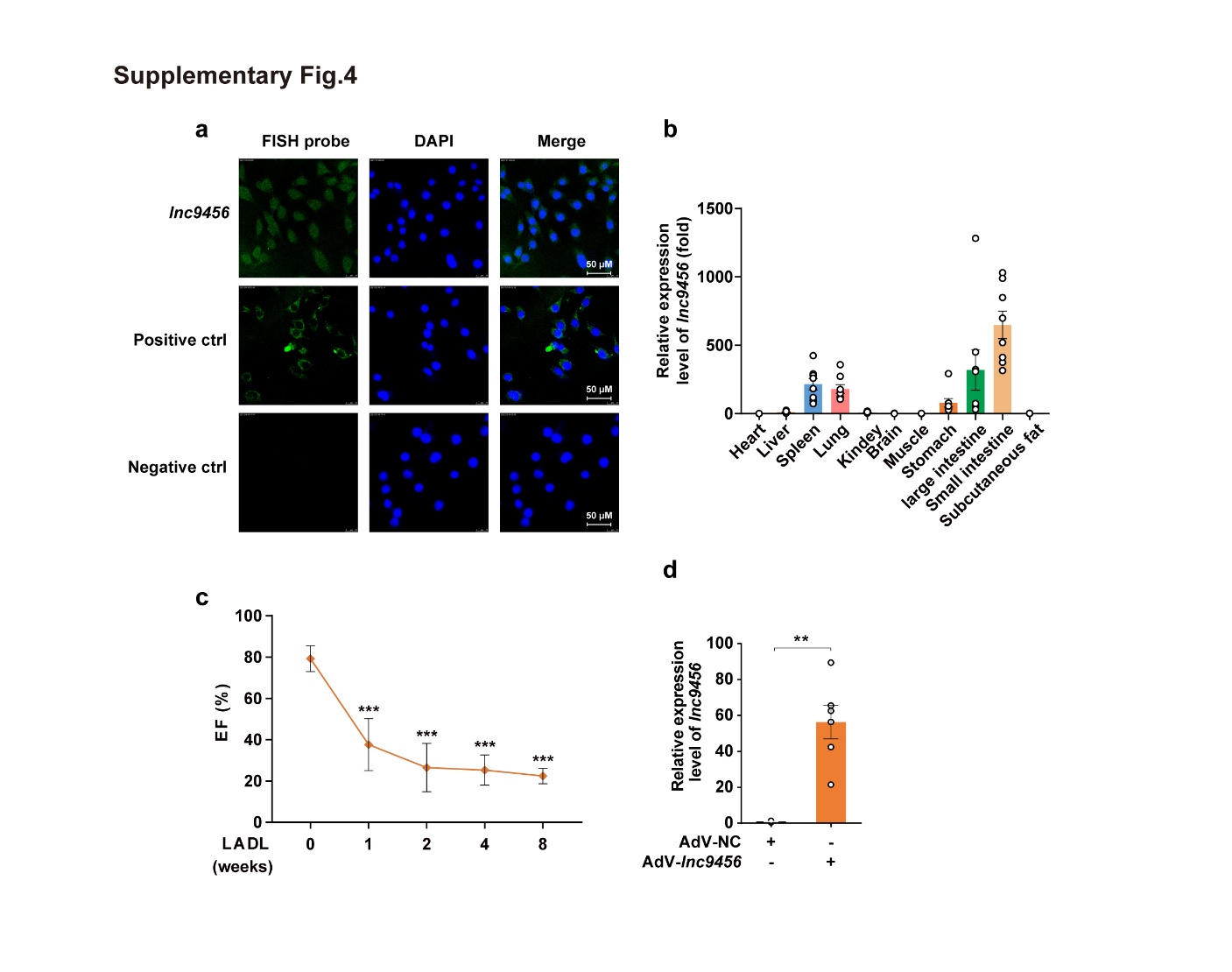


**Supplementary Fig. 4 Expression o**r **subcellular localization of the *lnc9456* in tissues or cardiomyocytes.**

**a** Representative images of FISH assay for the subcellular localization of *lnc9456* (green). The cell nuclei were stained with DAPI (blue). **b** qRT-PCR detection for *lnc9456* in the tissues of normal mice (n=8). **c** Echocardiographic analysis of EF (%) of mice after LADL surgery was evaluated at the indicated time points (n=6-10). **d** qRT-PCR detection for *lnc9456* of HL-1 mouse cardiomyocytes transfected with control adenovirus (AdV-Vector) or *lnc9456* overexpression adenovirus (AdV-*lnc9456*) for 48 h (n=5-6). Data are presented as the means ± SEM. **P<0.01, ***P<0.001 by one-way ANOVA with Dunnett’s multiple comparisons test (**c**) and Student’s t-test (**d**).

**
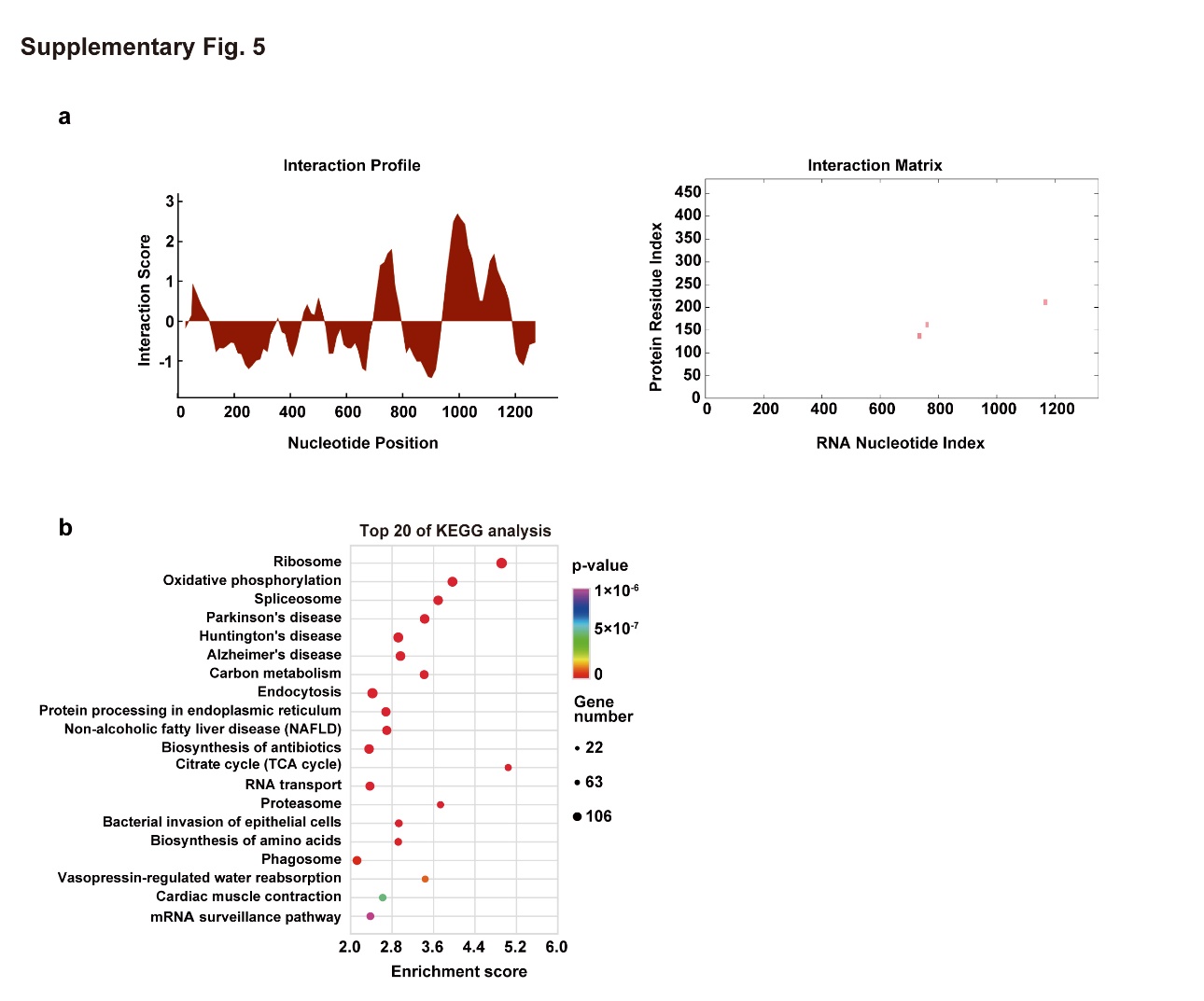
**

**Supplementary Fig. 5 Information of prediction and KEGG analysis for G3BP2 binding to *lnc9456*.**

**a** CatRAPID fragments module prediction of the interaction profile and matrix between G3BP2 and *lnc9456*. **b** The top 20 of KEGG analysis of interacting proteins pulled down by biotinylated *lnc9456* probe and identified by mass spectrometry.

**
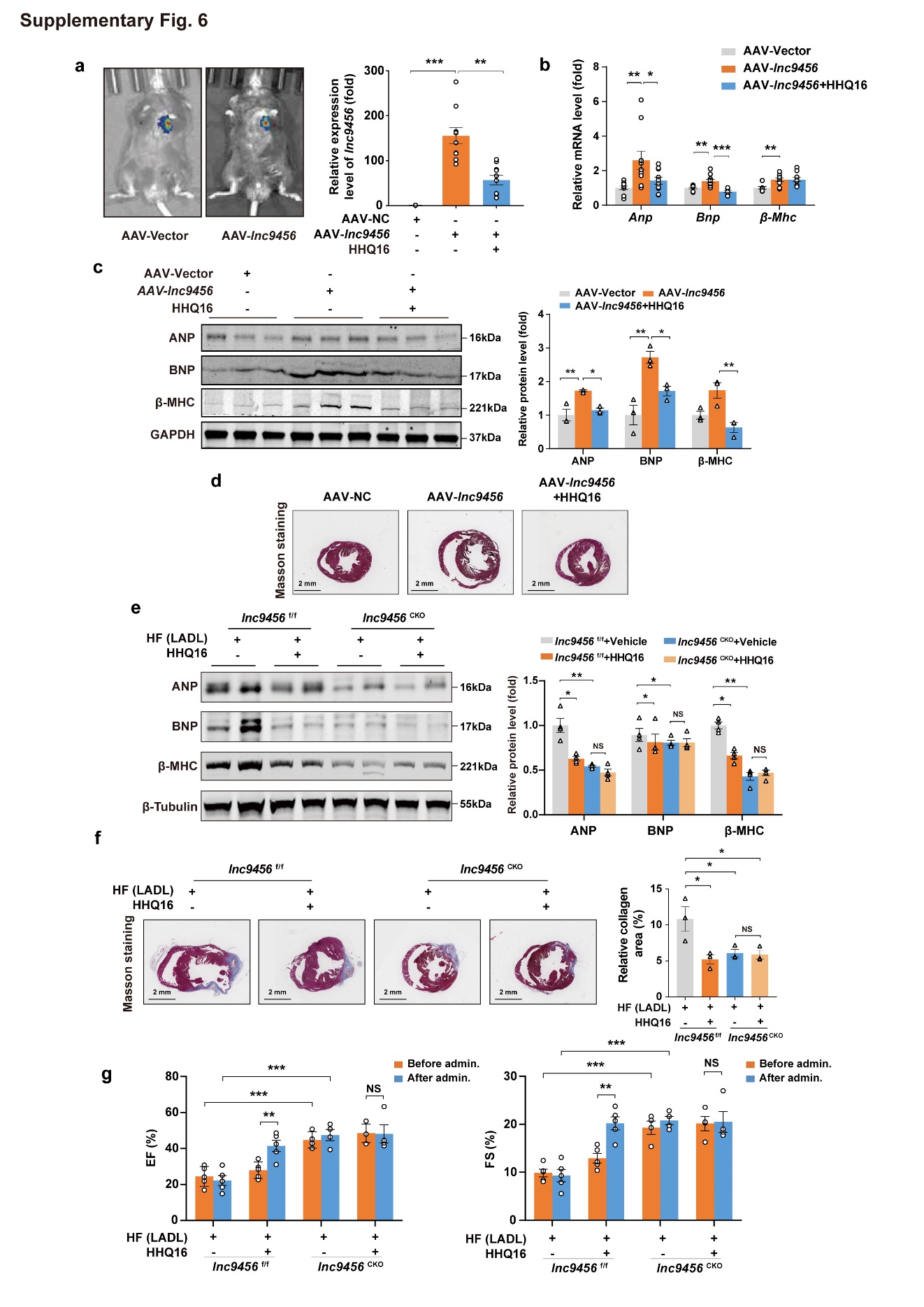
**

**Supplementary Fig. 6 Effects of HHQ16 on mouse heart when cardiomyocytes-specific overexpression or knockout of *lnc9456*.**

**a** Representative of *in vivo* imaging (left) and qRT-PCR detection of *lnc9456* (right) in myocardial tissues of mice. These mice were heart orthotopically injected with cardiomyocyte-specific *lnc94546* overexpression adeno-associated virus (AAV-*lnc9456*) driven by the *cTnT* promoter or its negative control (AAV-Vector). At 4^th^ week post injection, AAV-*lnc9456* mice were treated with 10 mg/kg of HHQ16 for 2 weeks, and the *in vivo* imaging of heart was captured and myocardial tissues were harvested (n=10). **b** qRT-PCR detection for *Anp*, *Bnp* and *β-Mhc* in myocardial tissues of mice treated as mentioned in Fig. 7**a** (n=10). **c** Western blotting (left) and its quantification (right) for ANP, BNP and β-MHC in the myocardial tissues derived from mice treated as mentioned in Fig. 7**a** (n=3). **d** Representative Masson's trichrome staining of the myocardial tissues derived from mice treated as mentioned in Fig. 7**a** (n=3). **e** Western blotting (left) and its quantification (right) for ANP, BNP and β-MHC in the myocardial tissues derived from *lnc9456*^f/f^ or *lnc9456*^CKO^ mice treated as mentioned in Fig. 7**f** (n=3). **f** Representative Masson's trichrome staining (left) and the quantification (right) of myocardial tissues derived from mice treated as mentioned in Fig. 7**f** (n=3). **g** Statistical analysis of EF (%) and FS (%) from *lnc9456*^f/f^ or *lnc9456*^CKO^ littermate female mice treated as mentioned in Fig. 7**f** (n=4-5). Data are presented as the means ± SEM. *P<0.05, **P<0.01, ***P<0.001 by one-way ANOVA with Tukey’s multiple comparisons test (**b, c, e, and f**) and two-way ANOVA with Sidak’s multiple comparisons test (**g**).

**
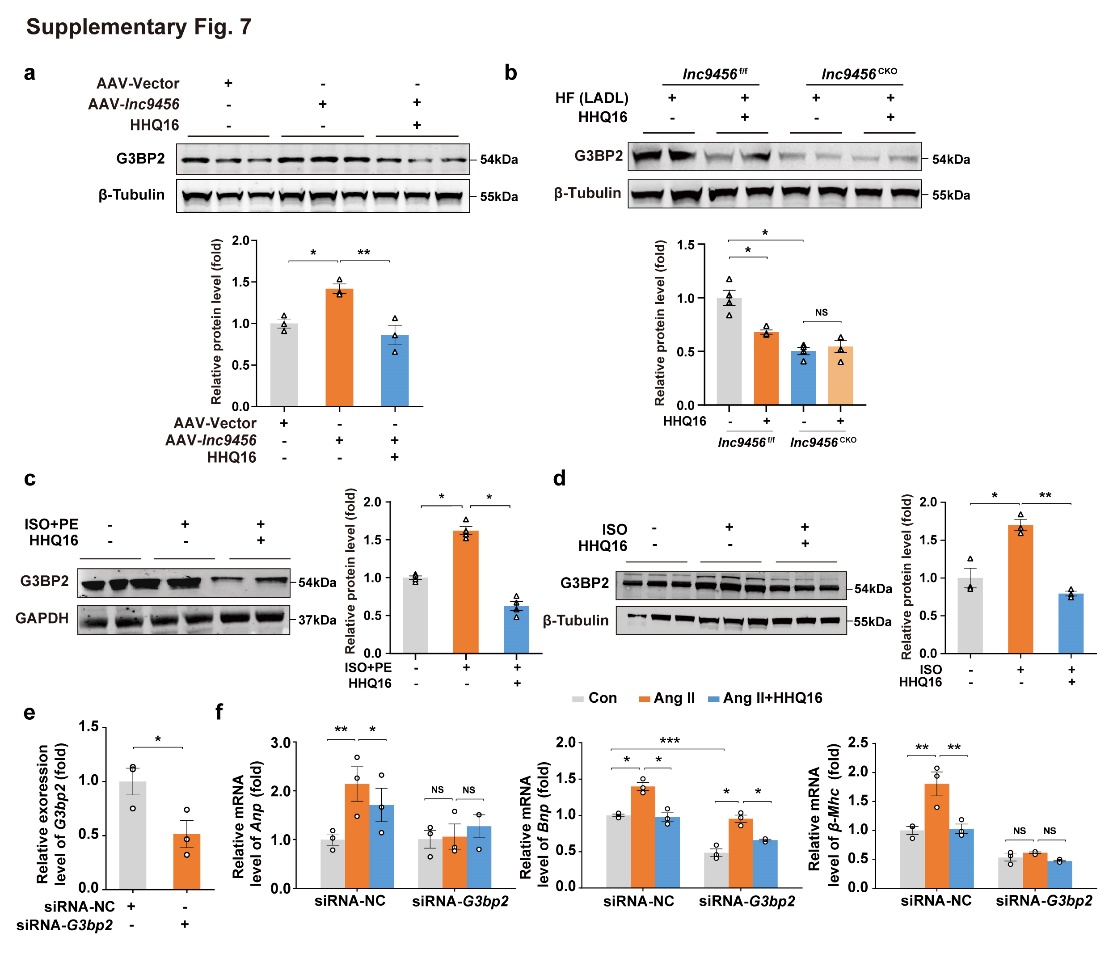
**

**Supplementary Fig. 7 G3BP2 mediates the effect of HHQ16 on cardiac hypertrophy.**

**a** Western blotting (upper) and its quantification (lower) for G3BP2 in myocardial tissues of mice treated as mentioned in Fig. 7**a** (n=3). **b** Western blotting (upper) and its quantification (lower) for G3BP2 in the myocardial tissues derived from *lnc9456*^f/f^ or *lnc9456*^CKO^ mice treated as mentioned in Fig. 7**f** (n=3). **c** Western blotting (upper) and its quantification (lower) for G3BP2 in HL-1 mouse cardiomyocytes treated with vehicle or ISO+PE (100 μM each) in the absence or presence of HHQ16 (100 nM) for 24 h (n=4). **d** Western blotting (upper) and its quantification (lower) for G3BP2 in neonatal mouse primary cardiomyocytes treated with vehicle or ISO (100 μM) in the absence or presence of HHQ16 (100 nM) for 24 h (n=3). **e** qRT-PCR detection for *G3bp2* in HL-1 cardiomyocytes transfected with control siRNA (siRNA-NC) or *G3BP2* siRNA (siRNA-*G3BP2*) for 72 h (n=3). **f** qRT-PCR detection for *Anp*, *Bnp*, and *β-Mhc* in HL-1 cardiomyocytes transfection with *G3BP2* siRNA (siRNA-*G3BP2*) or its negative control (siRNA-NC) for 48 h, then treated with vehicle or ISO+PE (100 μM each) in the absence or presence of HHQ16 (100 nM) for another 24 h (n=3). Data are presented as the means ± SEM. *P<0.05, **P<0.01, ***P<0.001 by one-way ANOVA with Tukey’s multiple comparisons test (**a-c**), Student’s t-test (**d**) and two-way ANOVA followed by Bonferroni’s post-hoc t-test (**f**).

**
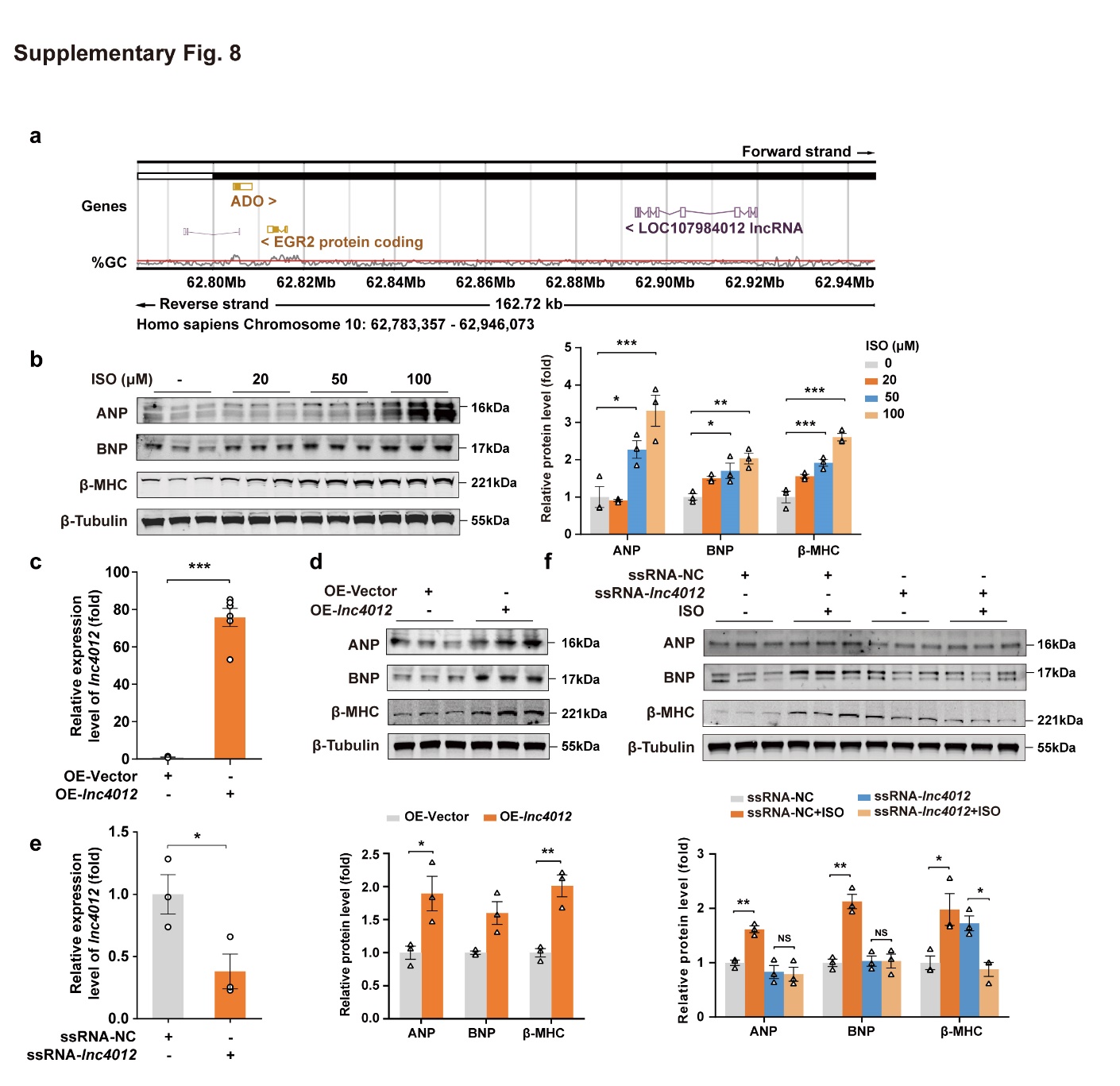
**

**Supplementary Fig. 8 Genomic locus, and the function of *lnc4012* in cardiomyocytes.**

**a** Genomic locus of the *lnc4012* and its adjacency to the *Egr2* gene from the Ensemble database. **b** Western blotting (left) and its quantification (right) for hypertrophic biomarkers ANP, BNP and β-MHC of AC16 human cardiomyocyte treated with vehicle or ISO (20, 50, 100 μM) for 24 h (n=3). **c** qRT-PCR detection for *lnc4012* of AC16 human cardiomyocytes transfected with control plasmid (OE-Vector) or *lnc4012*-overexpression plasmid (OE-*lnc4012*) for 48 h (n=6). **d** Western blotting (upper) and its quantification (lower) for ANP, BNP and β-MHC of AC16 human cardiomyocyte treated as mentioned in (**c**) (n=3). **e** qRT-PCR detection for *lnc4012* of AC16 human cardiomyocytes transfected with smart silencer RNA of *lnc4012* (ssRNA-*lnc4012*) or its negative control (ssRNA-NC) for 72 h (n=3). **f** Western blotting (upper) and its quantification (lower) for ANP, BNP and β-MHC in AC16 human cardiomyocyte transfected with smart silencer RNA of *lnc4012* (ssRNA-*lnc4012*) or its negative control (ssRNA-NC) for 48 h and then treated with vehicle or ISO (50 μM) for another 24h (n=3). Data are presented as the means ± SEM. *P<0.05, **P<0.01, ***P<0.001 by one-way ANOVA with Tukey’s multiple comparisons test (**b, f**) and Student’s t-test (**c**-**e**).

**
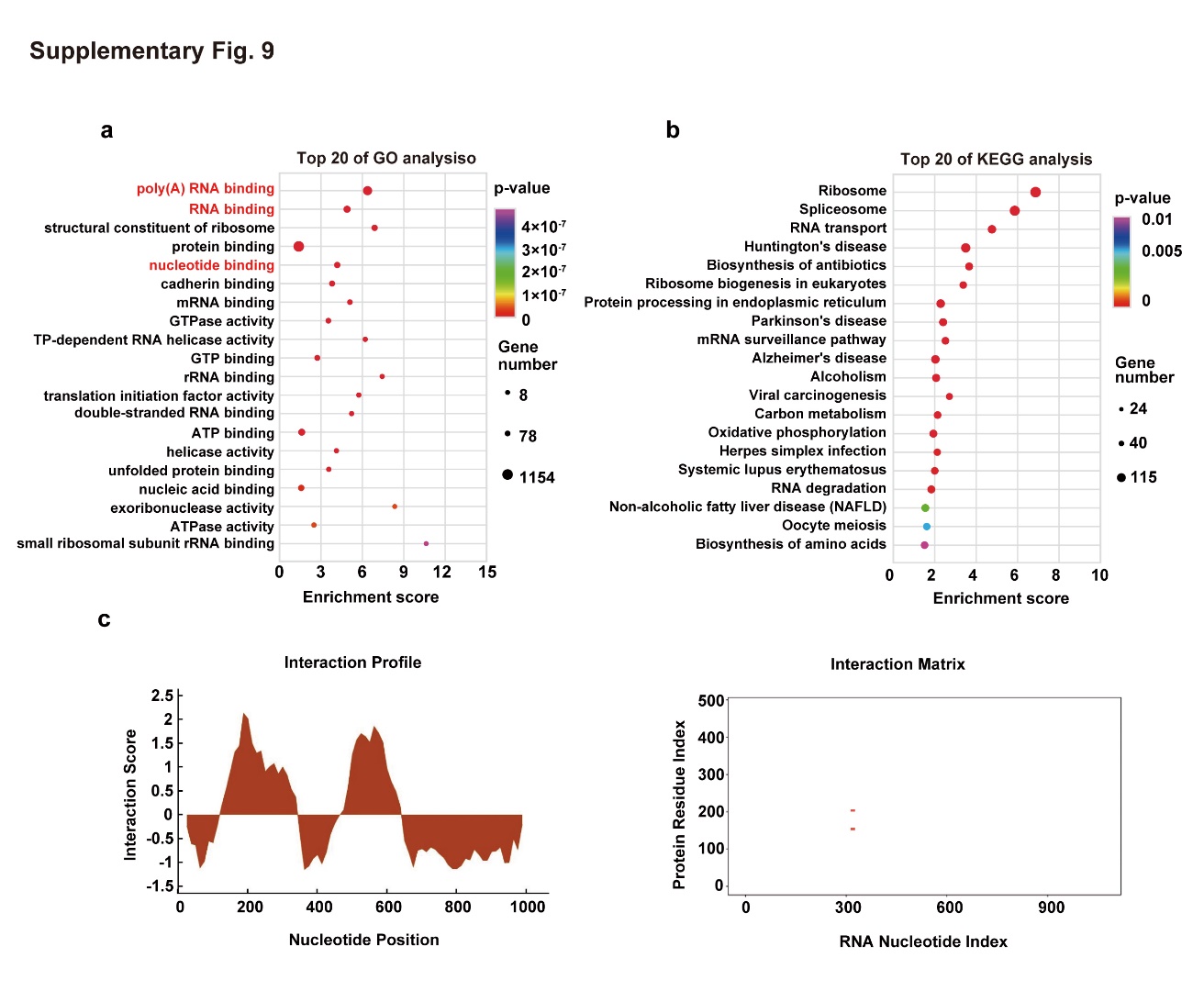
**

**Supplementary Fig. 9 Information of prediction, GO and KEGG analysis for G3BP2 binding to *lnc4012*.**

**a, b** The top 20 of GO (**a**) and KEGG (**b**) analysis of interacting proteins pulled down by biotinylated *lnc4012* probe and identified by mass spectrometry. **c** CatRAPID fragments module prediction of the interaction profile and matrix between G3BP2 and *lnc4012*, and a fragment, 351-378 bp of the transcript was identified as essential for the binding.

Supplementary Table 1. Sequence analysis obtained by RACE experiment of Gm32255 gene.

| Gene sequence |
| --- |
| GTGACACATCTGGAGATTTTCCACTGCCTGAAAGCTGCCTCGCGCCTAACCATGTTTTTCTATGGGTCTCGTGTGAAGAAGAATCTCCATTGGGTGAACGAGTTGACAGCGTTGCATGAGAACCTTGGCGTCTGATATTCACACCTGCTTGTGAAACAAACTACCGAGTGTTTCAATTACCCTCTGACCTTAGAAAAATGGAGGCTTCTAATTTGAGCTGTTGCTAGTCTTAAGCAAACAGCTTCTGCAGTTTTACACAACACTCTTGCTAGCCAAAGGAAGAAAAGTGTTGAGGTTTTTTTTTTCCCCCTGTGTGCAAAAGTTAGAAACAATTTTCACTTTCATTTCAGATTATGAAAGCCGAGTCACAGCCATAGCCACCCTCATTGAGATCTAGTTCTACCTGCATCGGCAGGGTTACTCTTCATGTGCGTCAGAGTAGAACCCATCACTGGTTCTGGATTCCAGTGGAAAGGTATGAATTAAAGTAAGCAGGGGCCATAGCGATGTGGCCAGCAAAGTTCTTGCTCAACAAGCATGCTCACCAAGTTCAAAGCCAACACCTATATTTAAAAAACTGGGACCAAGCATTCTGGAGCATGACTTTAACCCCTATACTCAGAAGATAGAGGTAGATGTTATCTCTGTGAGTTTAAGGTCAACCTGCTCTACATATGGAGTTCCAGGACAACCCGGGACTACATAGAGGGACCCTGCCTCAGACAAACAGAAATAACGACAAACAAGACATGCCAGCCCTGGGGCTGGAGAGATGAGTCTTCAGTTTAGAGCACGGGCCTCTCTCACATAGGACTTGGGTTCCATTCTAGCATCACTACGGCAGCTTACAACCACCTGTAACTCTAGTTCCAGGGACTCTGACGCTCTCTCCTGGCTCCTGCAGTAACCAGGATCACATGTAGTACACATGTATACATGTAGGCAAAACATTCATAAAGTAAGCAAATGATTTGTAAAATTCAAAGTGAGTCGTCAGAGTCCTGTGAACCTGTAGGGTCAGTGTTGGAGAGGCAGAGATAGGTTAGACCCTGGGTCTTGGTAGGTAGCTGGTCTAGCCAACACAGGAAGCTCCTGACTTATCGAGAGAGCCTGTCTCAATGCATAAGAGGGGTGGGTACTGCCAAAATGGCCGATCAGGTAAAGGGGTTTGTCACCAAGCCTGAGGGCCTGCGCTGGATCCCCAAGACACACATGATGGAAGAAGAGAAACAGCCCCCCCCCCCCAAGTTATCCTCTGACTACCCTATAGCATGAGTACACCTACATTTATTCATGTACAAGCAAATAAATATAATAAACAATTTTAATAAAGAAAAAGGATGGAAACTAAAAAAAAAAAAAAAA |
